# Integrated plasma proteomic and single-cell immune signaling network signatures demarcate mild, moderate, and severe COVID-19

**DOI:** 10.1016/j.xcrm.2022.100680

**Published:** 2022-06-28

**Authors:** Dorien Feyaerts, Julien Hédou, Joshua Gillard, Han Chen, Eileen S. Tsai, Laura S. Peterson, Kazuo Ando, Monali Manohar, Evan Do, Gopal K.R. Dhondalay, Jessica Fitzpatrick, Maja Artandi, Iris Chang, Theo T. Snow, R. Sharon Chinthrajah, Christopher M. Warren, Richard Wittman, Justin G. Meyerowitz, Edward A. Ganio, Ina A. Stelzer, Xiaoyuan Han, Franck Verdonk, Dyani K. Gaudillière, Nilanjan Mukherjee, Amy S. Tsai, Kristen K. Rumer, Danielle R. Jacobsen, Zachary B. Bjornson-Hooper, Sizun Jiang, Sergio Fragoso Saavedra, Sergio Iván Valdés Ferrer, J. Daniel Kelly, David Furman, Nima Aghaeepour, Martin S. Angst, Scott D. Boyd, Benjamin A. Pinsky, Garry P. Nolan, Kari C. Nadeau, Brice Gaudillière, David R. McIlwain

**Affiliations:** 1 Department of Anesthesiology, Perioperative and Pain Medicine, Stanford University School of Medicine, Stanford, CA, USA; 2 Section Pediatric Infectious Diseases, Laboratory of Medical Immunology, Radboud Institute for Molecular Life Sciences, Nijmegen, the Netherlands; 3 Radboud Center for Infectious Diseases, Radboud University Medical Center, Nijmegen, the Netherlands; 4 Center for Molecular and Biomolecular Informatics, Radboud University Medical Center, Nijmegen, the Netherlands; 5 Department of Microbiology and Immunology, Stanford University School of Medicine, Stanford, CA, USA; 6 Division of Neonatal and Developmental Medicine, Department of Pediatrics, Stanford University School of Medicine, Stanford, CA, USA; 7 Sean N Parker Center for Allergy and Asthma Research, Stanford University, Stanford, CA, USA; 8 Department of Medicine, Stanford University, Stanford, CA, USA; 9 Department of Primary Care and Population Health, Stanford University School of Medicine, Stanford, CA, USA; 10 Division of Allergy, Immunology and Rheumatology, Department of Pediatrics, Stanford University, Stanford, CA, USA; 11 Department of Biomedical Sciences, University of the Pacific, Arthur A. Dugoni School of Dentistry, San Francisco, CA, USA; 12 Division of Plastic & Reconstructive Surgery, Department of Surgery, Stanford University School of Medicine, Stanford, CA, USA; 13 Departamento de Neurología, Instituto Nacional de Ciencias Médicas y Nutrición Salvador Zubirán, Mexico City, Mexico; 14 Plan de Estudios Combinados en Medicina (MD/PhD Program), Facultad de Medicina, Universidad Nacional Autónoma de México, Mexico City, Mexico; 15 Department of Epidemiology and Biostatistics, UCSF, San Francisco, CA, USA; 16 Institute for Global Health Sciences, UCSF, San Francisco, CA, USA; 17 F.I. Proctor Foundation, UCSF, San Francisco, CA, USA; 18 Buck Artificial Intelligence Platform, Buck Institute for Research on Aging, Novato, CA, USA; 19 Stanford 1000 Immunomes Project, Stanford University School of Medicine, Stanford, CA, USA; 20 Austral Institute for Applied Artificial Intelligence, Institute for Research in Translational Medicine (IIMT), Universidad Austral, CONICET, Pilar, Buenos Aires, Argentina; 21 Department of Biomedical Data Science, Stanford University School of Medicine, Stanford, CA, USA; 22 Department of Pathology, Stanford University School of Medicine, Stanford, CA, USA; 23 Division of Infectious Diseases and Geographic Medicine, Department of Medicine, Stanford University School of Medicine, Stanford, CA, USA; 24 Department of Medicine, Division of Pulmonary, Allergy and Critical Care Medicine, Stanford University, Stanford, CA, USA; 25 Department of Pediatrics, Stanford University, Stanford, CA, USA

**Keywords:** mass cytometry, SARS-CoV-2, COVID-19, proteomics, Olink, stacked generalization, CyTOF, immunophenotyping, PBMC, phosphosignaling response

## Abstract

The biological determinants underlying the range of coronavirus 2019 (COVID-19) clinical manifestations are not fully understood. Here, over 1,400 plasma proteins and 2,600 single-cell immune features comprising cell phenotype, endogenous signaling activity, and signaling responses to inflammatory ligands are cross-sectionally assessed in peripheral blood from 97 patients with mild, moderate, and severe COVID-19 and 40 uninfected patients. Using an integrated computational approach to analyze the combined plasma and single-cell proteomic data, we identify and independently validate a multi-variate model classifying COVID-19 severity (multi-class area under the curve [AUC]_training_ = 0.799, p = 4.2e-6; multi-class AUC_validation_ = 0.773, p = 7.7e-6). Examination of informative model features reveals biological signatures of COVID-19 severity, including the dysregulation of JAK/STAT, MAPK/mTOR, and nuclear factor κB (NF-κB) immune signaling networks in addition to recapitulating known hallmarks of COVID-19. These results provide a set of early determinants of COVID-19 severity that may point to therapeutic targets for prevention and/or treatment of COVID-19 progression.

## Highlights

• Proteomic analysis of plasma analytes and immune cells in blood during COVID-19
• Multi-variate stacked generalization LASSO model classifies COVID-19 severity
• Dampening of intracellular signaling responses with increasing COVID-19 severity
• Enrichment of plasma proteins with increasing severity

## Graphical abstract

**Figure:**
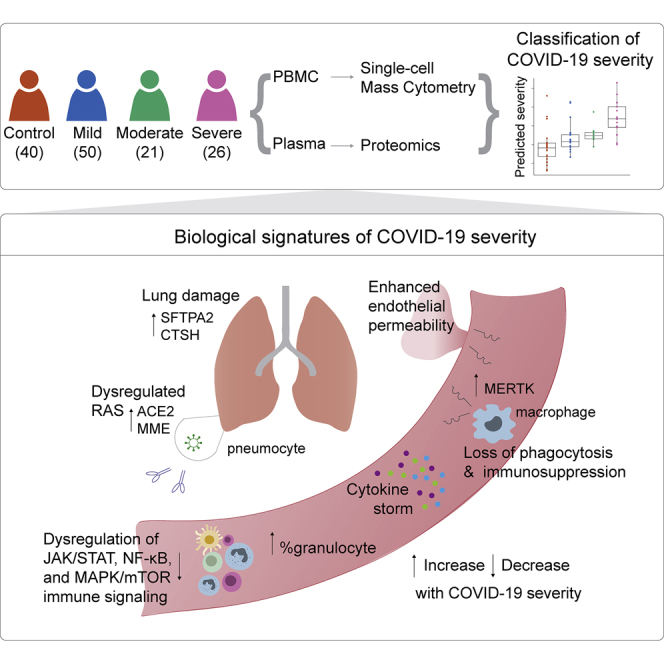

## Teaser

Feyaerts et al. integrate plasma and single-cell proteomic analysis of patients with mild, moderate, and severe COVID-19 to reveal biological signatures that differentiate COVID-19 severity, including dysregulation of pro-inflammatory signaling networks.

## Introduction

The ongoing coronavirus disease 2019 (COVID-19) pandemic, caused by the novel and highly contagious severe acute respiratory syndrome coronavirus 2 (SARS-CoV-2),^1^ has affected more than 240 million patients worldwide as of late 2021.^2^ A wide range of clinical manifestations exists for COVID-19, which require different intervention strategies. While the majority of patients with COVID-19 experience mild or asymptomatic infections, nearly 20% of patients develop severe disease requiring hospitalization.^3^ A substantial portion (8%–30%) of those hospitalized patients ultimately succumbs to the disease, leading to a devastating global tally of COVID-19 fatalities.^3^, ^4^, ^5^, ^6^, ^7^

Varying outcomes for COVID-19 depend on a set of risk factors and the interplay between viral replication and tissue damage, as well as a balance of beneficial and detrimental host immune responses. Several studies have provided evidence for profoundly altered immune responses caused by SARS-CoV-2 infection, including sustained functional changes in circulating immune cells. Lymphopenia,^8^, ^9^, ^10^, ^11^, ^12^ increased inflammatory plasma cytokine levels,^11^^,^^13^^,^^14^ dysregulated innate immune cell function,^15^, ^16^, ^17^, ^18^ and abnormal T cell activation and exhaustion^11^^,^^19^^,^^20^ have been observed, particularly in hospitalized patients with severe COVID-19. However, prior studies have primarily focused on patients with severe COVID-19, while fewer studies have included non-hospitalized patients with mild and moderate COVID-19.^18^^,^^21^^,^^22^ In addition, while prior studies have reported on the distribution, phenotype, and transcriptional profile of peripheral immune cells, how SARS-CoV-2 infection alters immune cell signaling responses to inflammatory challenges (or immune signaling networks) has not been determined. As such, the immunological mechanisms that differentiate patients with mild, moderate, and severe COVID-19 are poorly understood. Unraveling the underlying immune pathogenesis across the spectrum of COVID-19 presentations is important to both understand the drivers of disease severity as well as to identify clinically relevant biological signatures that could inform therapeutic interventions.

High-dimensional mass cytometry immunoassays are uniquely adapted to the analysis of immune cell signaling networks as multiple intracellular signaling events (e.g., post-translational protein modifications) are simultaneously quantified in precisely phenotyped immune cells in their endogenous state and in response to *ex vivo* stimulations.^23^ The approach has previously enabled the identification of clinically relevant biological signatures predictive of patient outcomes in several clinical contexts, including infection, immunization, malignancies, stroke, and traumatic injury.^24^, ^25^, ^26^, ^27^, ^28^, ^29^, ^30^, ^31^

In this cross-sectional study, we combined the mass cytometry analysis of immune cell signaling responses with the high-content proteomic analysis of plasma analytes in blood samples from patients identified with mild, moderate, and severe COVID-19 to establish biological signatures that demarcate COVID-19 clinical manifestations. The integrated single-cell and plasma proteomic analysis allowed including an additional dimension in the characterization of immune signaling networks by accounting for the plasma environment of circulating immune cells.

## Results

### Combined plasma and single-cell proteomic analysis of peripheral blood samples from patients with mild, moderate, and severe COVID-19

**Figure 1:**
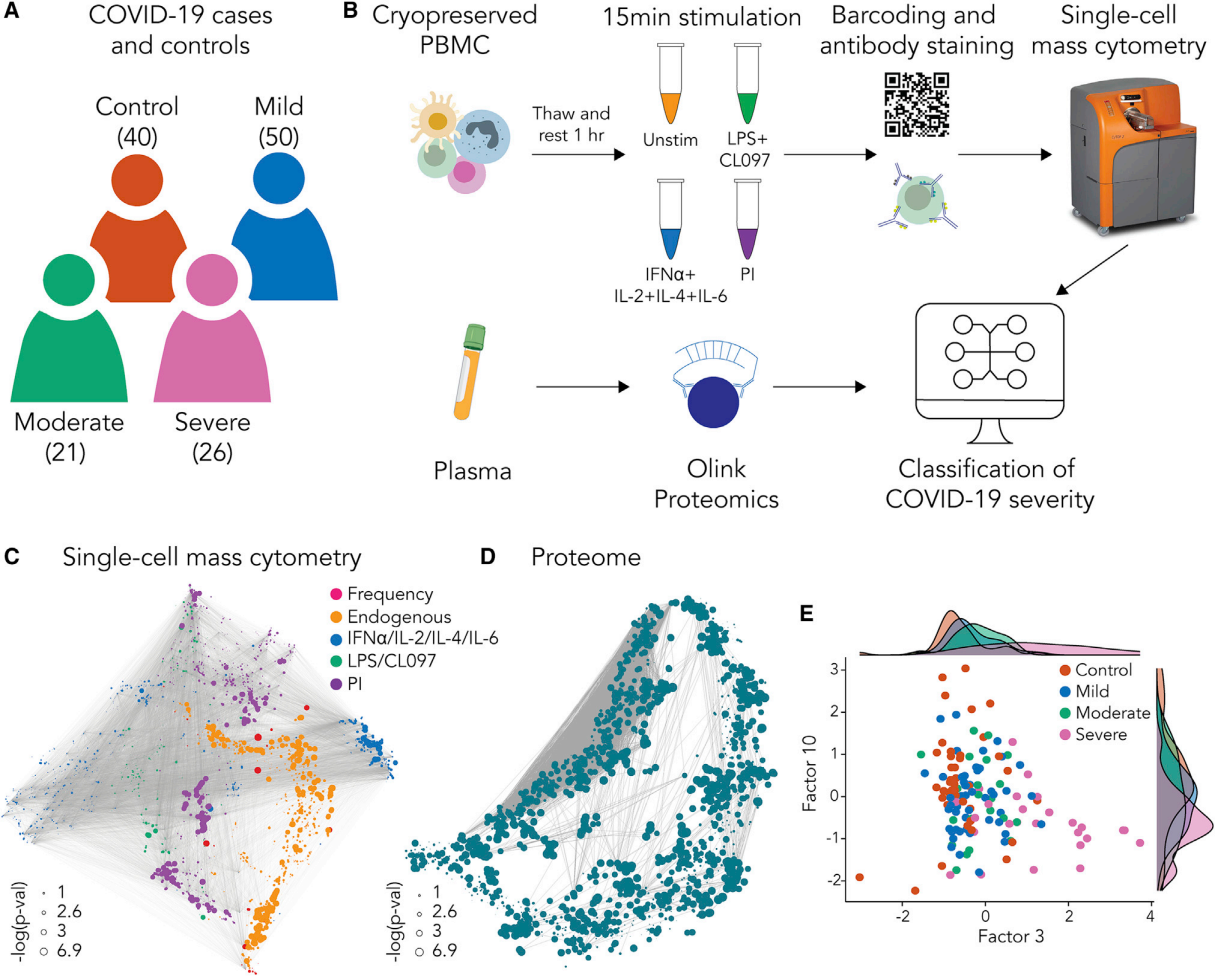
Combined plasma and single-cell proteomic profiling of patients with mild, moderate, and severe COVID-19 (A) Patients with mild (n = 50), moderate (n = 21), and severe (n = 26) COVID-19 were examined together with healthy controls (n = 40). (B) Schematic representation of the experimental workflow. Plasma proteins were measured using the Olink Explore 1536 assay, while PBMCs were stimulated with either LPS + CL097, IFNα + IL-2 + IL-4 + IL-6, PMA + ionomycin (PI), or left unstimulated (Unstim) for endogenous signaling before barcoding, antibody staining, and analysis by single-cell mass cytometry. (C and D) Correlation networks of single-cell mass cytometry and proteome dataset. Each node represents a feature, with edges representing the correlation between features (cor > 0.9). Node size reflects -log10 of p value of the correlation with severity (Spearman), and node color represents the different data layers. (E) Bivariate scatterplot of patients with COVID-19 and healthy controls plotted along factors 3 and 10 identified by multi-omics factor analysis (MOFA; see also Figure S3).

Ninety-seven SARS-CoV-2-positive patients with mild, moderate, or severe COVID-19 were enrolled in this cross-sectional study at Stanford University Medical Center (CA, USA) (Figure 1A). Patient characteristics can be found in Table 1. COVID-19 severity was determined using previously defined clinical National Institute of Health (NIH) criteria and assigned at the time of a SARS-CoV-2 qRT-PCR test^32^^,^^33^ (see STAR Methods). In brief, SARS-CoV-2-positive patients categorized as mild had zero or mild COVID-19 symptoms without any breathing issues. Moderate patients had signs of lower respiratory tract disease with an oxygen saturation above 94%. All severe patients were hospitalized due to respiratory distress. Importantly, none of the patients had received COVID-19 treatment at time of sample collection. Samples from SARS-CoV-2-positive patients were examined alongside those from 40 healthy controls collected at Stanford in 2019, before the detection of SARS-CoV-2 in the geographic region.

**Table 1:** Patient characteristics

	Control (N = 40)		Mild (N = 50; 2 asymptomatic)		Moderate (N = 21)		Severe (N = 26)	
Age	48 (23–73)		41.5 (23–78)		45 (19–78)		52.5 (29–78)	
Gender	N	%	N	%	N	%	N	%
M	16	40	23	46	8	38	12	46.2
F	24	60	27	54	13	62	14	53.8
Race	N	%	N	%	N	%	N	%
Asian	11	27.5	12	24	2	9.5	2	7.7
Hispanic/Latino	0	0	5	10	3	14.3	12	46.2
White	15	37.5	23	46	10	47.6	4	15.4
Black	1	2.5	3	6	0	0	3	11.5
Multi-racial	3	7.5	0	0	1	4.8	0	0
Middle Eastern	1	2.5	0	0	0	0	0	0
Not reported	9	22.5	7	14	5	23.8	5	19.2
HispanicRace/Ethnicity	N	%	N	%	N	%	N	%
Yes	0	0	9	18	4	19	14	53.8
No	31	77.5	34	68	13	61.9	7	26.9
Not reported	9	22.5	7	14	4	19	5	19.2
Comorbidities								
Diabetes	N	%	N	%	N	%	N	%
Yes	0	0	4	8	1	4.8	9	34.6
Prediabetic	1	2.5	3	6	1	4.8	0	0
No	32	80	34	68	15	71.4	14	53.8
Not reported	7	17.5	9	18	4	19	3	11.5
Asthma	N	%	N	%	N	%	N	%
Yes	4	10	7	14	5	23.8	8	30.7
No	29	72.5	33	66	12	57.1	11	42.3
Not reported	7	17.5	10	20	4	19	7	26.9
CV Condition	N	%	N	%	N	%	N	%
Yes	5	12.5	2	4	1	4.8	7	26.9
No	28	70	48	96	20	95.2	19	73.1
Not reported	7	17.5	0	0	0	0	0	0
Obesity	N	%	N	%	N	%	N	%
Yes	0	0	3	6	2	9.5	13	50
No	33	82.5	32	64	14	66.7	10	38.5
Not reported	7	17.5	15	30	5	23.8	3	11.5
Outcome, death	0	0	0	0	0	0	3	11.5
Days between self-reported symptom onset and sample collection			25.5 (-1–69)		22 (-1–66)		5 (0–75)	

Data are shown as a number and a percentage. Age is reported in median years (minimum to maximum), and the days between onset of self-reported COVID-19 symptoms and sample collection is reported in median days (minimum to maximum). The category multiracial includes the variables Multiracial, Asian/Native American, White/Asian/Pacific Islander, and/or Mexican/Native American. The category White includes White, White/Hispanic, and White/non-Hispanic. The category Black includes both Black and Black/Hispanic/Latino. The category Hispanic/Latino includes the variables Hispanic, Hispanic/Latino, and Mexican/Hispanic. Ethnicity makes the distinction between those who reported as Hispanic and those who did not.

Blood samples were used to isolate both plasma and peripheral blood mononuclear cells (PBMCs). PBMCs were stimulated *ex vivo* to trigger pathogen sensing and cytokine signaling response pathways in innate and adaptive immune cells relevant during infection (TLR4 stimulant lipopolysaccharide [LPS] and TLR7/8 agonist CL097, interferon alpha [IFNα], interleukin-2 [IL-2], IL-4, and IL-6 cytokine cocktail, and cell stimulation cocktail consisting of phorbol 12-myristate 13-acetate [PMA] and ionomycin [I] [PI]) (Figure 1B). The frequencies of 44 manually gated immune cell subsets representing major circulating innate and adaptive immune cells were determined using a 42-parameter single-cell phospho-mass cytometry immunoassay (Figure S1). For each immune cell subset, the frequency was determined, alongside endogenous signaling activity (unstimulated condition) and signaling response capacity of cells to *ex vivo* stimulation with inflammatory reagents, which were measured by examining the phosphorylation state of 15 intracellular signaling proteins and 5 markers that relate to immune-subset-specific functionality (see STAR Methods and Figure S2).^23^ After penalization, the mass cytometry analysis generated a total of 2,662 immune cell response features per PBMC sample. To complement this single-cell analysis, 1,472 circulating plasma proteins were measured using the proximity extension assay (PEA) platform from Olink Proteomics. Five mass cytometry (cell frequency, endogenous signaling, IFNα/IL-2/IL-4/IL-6 signaling response, LPS/CL097 signaling response, and PI signaling response) and one plasma proteomic data layer(s) were collected, resulting in six data layers in total. Correlation networks demonstrate the existence of strong inter- and intra-layer correlations between features (Figures 1C and 1D).

To understand the relationships between the different data layers and COVID-19 severity, we first applied unsupervised dimensionality reduction with multi-omics factor analysis (MOFA^34^), which infers a set of factors that capture shared sources of variability across different datasets. We supplied all six data layers for the analysis, resulting in a MOFA model with 17 factors (Figure S3A, model trial 6). Both single-cell immune response (endogenous signaling and signaling response to IFNα/IL-2/IL-4/IL-6 and LPS/CL097; factor 10) and plasma proteome (factor 3) data contributed strongly to the variance observed in our samples (Figure S3B) and were significantly associated with COVID-19 severity (Figure S3C). A gradient with increasing disease severity across factors 3 and 10 (Figure 1E) was also observed, suggesting that single-cell immune response and plasma proteome data both contain clinically important biological events. The results prompted us to perform an integrated analysis to determine whether a classifier of COVID-19 severity could be derived from the combined plasma and single-cell proteomics data.

### Integrated modeling of plasma and single-cell proteome differentiates COVID-19 severity

**Figure 2:**
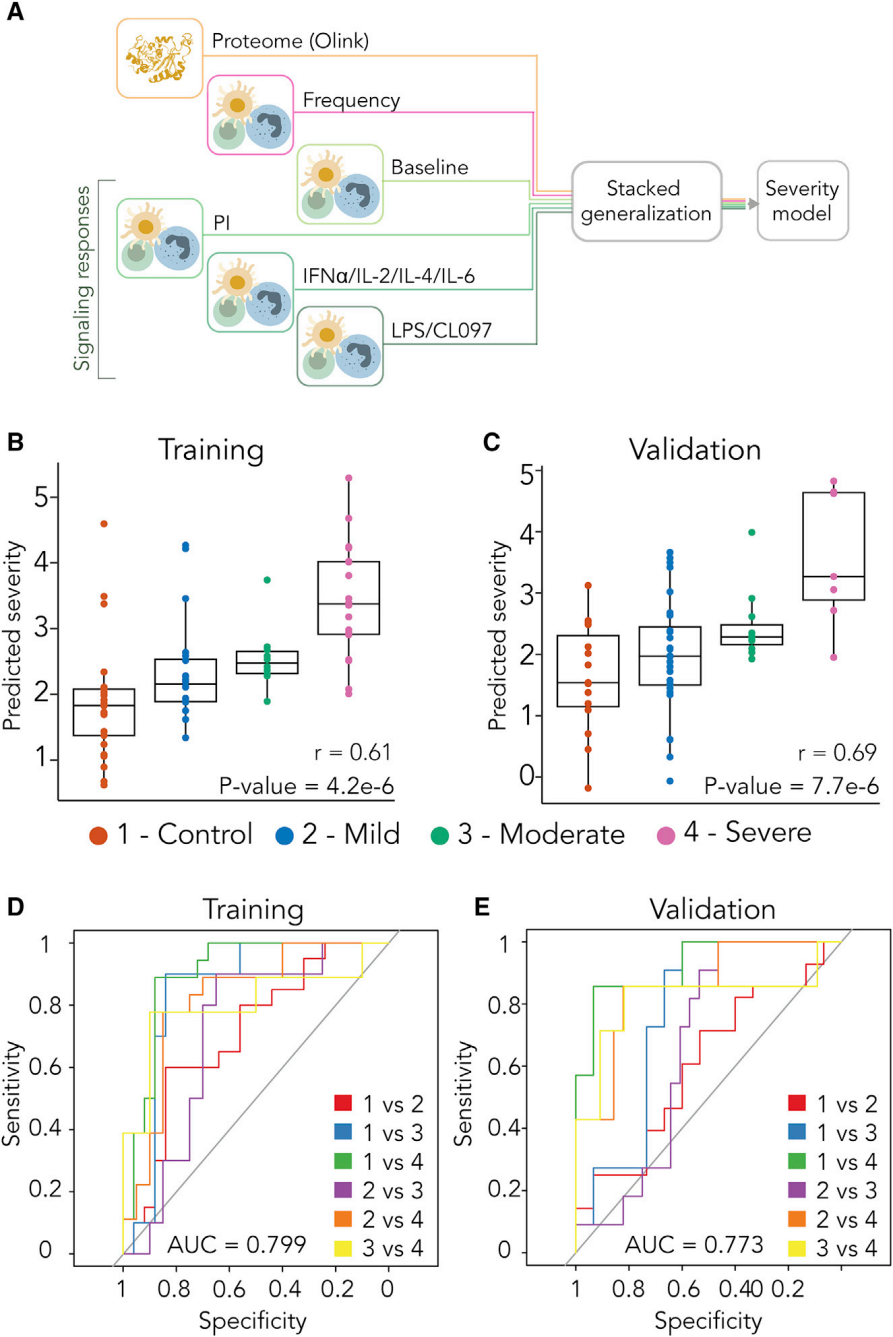
Integrated modeling of plasma and single-cell proteomic events categorizes COVID-19 severity (A) LASSO linear regression models were trained for each individual data layer before integration of all six data layers (proteome, frequency, endogenous signaling, LPS/CL097 signaling response, IFNα/IL-2/IL-4/IL-6 signaling response, and PI signaling response) using a stacked generalization (SG) method. (B and C) Outcome of predicted versus true disease severity derived from SG model for the (B) training (r = 0.61, p = 4.2e-6, n = 74) and (C) validation cohort (r = 0.69, p = 7.7e-6, n = 73). (D and E) Multi-class area under the curve receiver operating characteristic (ROC) analysis of the training (D; AUC = 0.799, n = 74) and validation (E; AUC = 0.773, n = 63) severity model (see Table S2 for individual AUCs). 1 = control, 2 = mild, 3 = moderate, and 4 = severe. For boxplots, the center line represents the median value; upper and lower box limits indicate first (Q1) and third (Q3) quartile, respectively; whiskers, minimum (Q1−1.5∗IQR) and maximum (Q3+1.5∗IQR). IQR, interquartile range. AUC, area under the curve. See also Figure S6 and Tables S1–S4.

A high-dimensional computational analysis pipeline was applied to train and independently validate (training cohort: n = 74; 25 control, 20 mild, 10 moderate, and 19 severe patients; validation cohort: n = 63; 15 control, 30 mild, 11 moderate, and 7 severe patients) an integrated model of COVID-19 severity based on the combined proteomic and single-cell immune response data.^35^ In this approach, the six data layers were considered separately, and a two-step process was used to combine these data layers in a multi-omic fashion (Figure 2A). Cross-validated multi-variate least absolute shrinkage and selection operator (LASSO) linear regression models^36^ were first trained for each individual data layer of the training cohort, with disease severity used as a ranked order variable (i.e., control classed as 1 to severe classed as 4), and second, the individual LASSO models were integrated into a single model by stacked generalization (SG)^35^ (Figure 2A). The second step uses the estimations of disease severity of each LASSO model as predictors for a constrained regression model.

The analysis identified an SG model (“severity model”) that classified COVID-19-severity categories at time of sampling for patients in the training cohort (r = 0.61, p = 4.2e-6, n = 74). The generalizability of the severity model was independently tested in patients from the validation cohort (r = 0.69, p = 7.7e-6, n = 63) (Figures 2B and 2C). The contribution of individual data layers to the overall severity model was highest for the plasma proteome and lowest for the signaling responses to LPS/CL097 stimulation, according to severity model coefficients (Table S1).

To estimate the performance of the severity model, a multi-class area under the curve receiver operating characteristic (ROC) analysis was performed for the training and validation cohort (Figures 2D and 2E). The multi-class ROC analysis showed that the severity model performed well at classifying patients across disease-severity categories (multi-class area under the curve [AUC]_training_ = 0.799; multi-class AUC_validation_ = 0.773).^37^^,^^38^ The most accurate classification was achieved when classifying severe patients from the other patient groups (Table S2), with a model performance similar to the classifier of severe disease described by Filbin et al*.*^39^ In addition, different regression strategies were tested showing similar results, with the best overall performance obtained using LASSO regression (Table S3). Among patients for whom infection-related clinical laboratory parameters were available, model values for signatures of COVID-19 severity significantly correlated with both CRP and D dimer (Spearman correlation: CRP, r = 0.803, p = 0.0002; D dimer, r = 0.695, p = 0.0051).

Results from the severity model indicate that mild, moderate, and severe COVID-19 manifestations can be differentiated from the measurement of plasma proteins and single-cell immune signaling events in patient’s peripheral blood. Several clinical and (socio)demographic variables such as old age, male gender, high BMI, and Hispanic ethnicity have previously been shown to be risk factors for COVID-19 severity.^12^^,^^39^, ^40^, ^41^, ^42^, ^43^, ^44^ In addition, recent studies have highlighted the dynamic changes of innate and adaptive immune responses over the course of COVID-19 disease,^9^^,^^18^ such that the timing of sample collection (Figure S4) may affect the detection of immune features related to disease severity. We observed significant correlations between the covariates of obesity, Hispanic ethnicity, and days since symptom onset with COVID-19 severity (Figure S5). To account for inter-patient variability in these key clinical, (socio)demographic, and experimental variables, we performed a confounder analysis. The results showed that the SG model remained significantly predictive of COVID-19 severity when accounting for the variables age, gender, obesity, Hispanic ethnicity, and time between reported symptom onset and sample collection (Table S4).

Our SG severity model was built and independently tested in a cohort of patients recruited at a single center. A recent proteomic study^39^ of patients enrolled at the Mass General Hospital (MGH, Boston, MA, USA) that used an identical proteomic assay (the Olink Explore 1536) provided an opportunity to examine, at least partially, the generalizability of our findings to a broader patient population. A severity model built on the plasma proteomic data from the Stanford cohort accurately predicted the disease severity of patients included in the MGH cohort (Figure S6; r = −0.453541; p < 2e-16), providing independent validation of the plasma proteomic component of our predictive model in a second patient cohort. Notably, a significant correlation was found between the two studies for the set of 195 proteomic features associated with COVID-19 severity described in Filbin et al.^39^ (r = 0.40, p < 0.0001, Pearson, -log-adjusted p value; Data S1B).

### Biological signatures of COVID-19 severity

**Figure 3:**
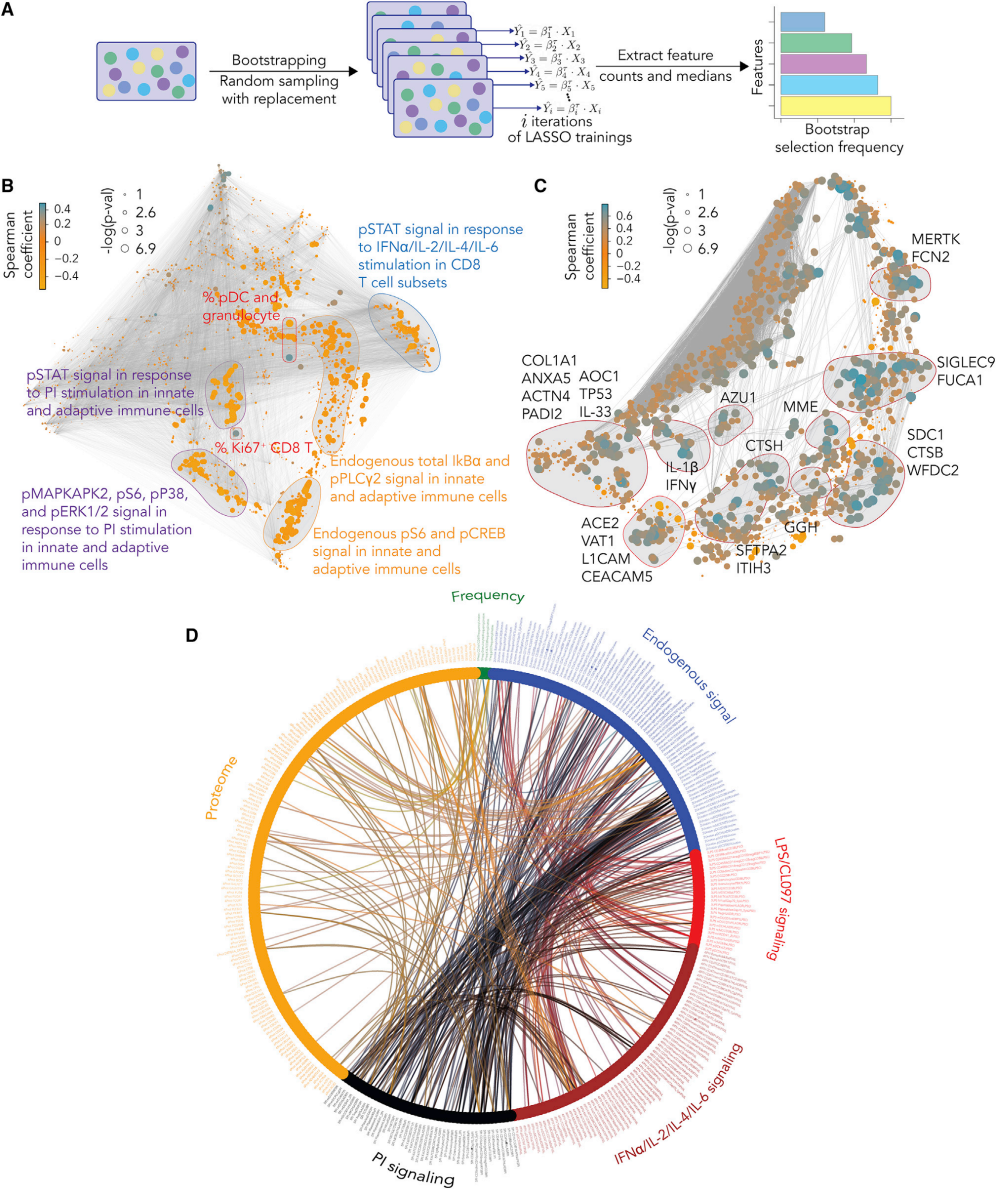
An iterative bootstrapping method identifies robust informative features for the differentiation of mild, moderate, and severe COVID-19 (A) Workflow of the iterative bootstrap method used to identify informative features in the six data layers of the severity model. LASSO regression model was run 1,000 times on random sub-samples with replacement for each data layer, X_i_, then the number of times an individual feature was selected in one of the bootstrap iterations was counted, and the features were ranked according to the frequency of selection in the bootstrap models. (B and C) Correlation network depicting single-cell (B) or plasma (C) proteomic features. Edges represent the correlation between features (Spearman cor > 0.9). Blue/orange nodes highlight positive/negative correlation with disease severity. Node size reflects -log10 of p value (Spearman). Communities containing the bootstrap-selected informative single-cell (B) or plasma (C) proteomic features are highlighted and annotated. (D) Interomic correlations between features of the six data layers are visualized in a chord diagram. Interomic correlations of the top 10% features ranked by bootstrap with absolute Spearman correlation coefficients between 0.5 and 1.0 are shown.

To facilitate biological interpretation of the high-dimensional severity model, the contribution of individual plasma and single-cell proteomic features to the severity model performance was quantified by measuring repeated selection during a 1,000-iteration bootstrap procedure^45^^,^^46^ (Figure 3). This bootstrap procedure recreates the dataset 1,000 times by sampling from the original dataset with replacement, and a new cross-validated LASSO model is trained for each iteration.^45^^,^^46^ The relative importance of each feature to the model is based on frequency of selection for a given feature (Data S1). We examined in detail the top 10% of total features ranked by the bootstrap procedure and that thus contributed strongly to the overall model (Figures 3B and 3C; Data S1).

Single-cell (Figure 3B) and plasma (Figure 3C) proteomic features were visualized with two correlation networks, highlighting the correlation between individual features (edges) and between individual features and COVID-19 severity (node size/color). Features within top 10% of bootstrap selection segregated into correlated communities, which were annotated according to the cellular attribute most commonly represented within each community (immune cell frequency or signaling response for single-cell features, Figure 3B; protein name for plasma proteomic features, Figure 3C). To complement the analysis of “intraomic” correlations within omic datasets, “interomic” correlations between features from different single cell or plasma proteomic data layers contributing the most to the severity model were visualized on a chord diagram (Figure 3D). The chord diagram highlighted multiple interomic correlations, 29% of which occurred between plasma proteome and single-cell proteomic features, with the most correlations between plasma proteome components and endogenous phosphorylated (p)S6 and pCREB signal in Ki67^+^ CD8 T cells and frequency of plasmacytoid dendritic cells (pDCs). Plasma proteins correlating with these cellular features were enriched for those involved in cytokine signaling (Reactome pathway identification). This analysis highlighted the interconnected nature of single-cell and plasma proteomic features of the severity model and underscored the need for an integrated approach to characterize the inflammatory state of patients with varying COVID-19 severity.

With respect to cell frequency features contributing the most to the severity model, we observed changes in immune cell distribution that are reminiscent of recent immunophenotyping studies in patients with severe COVID-19. For instance, in our study and prior reports, pDCs and CD161^+^CD8^+^ T cell frequencies were negatively correlated, while Ki67^+^CD8^+^ T cell and granulocyte frequencies were positively correlated with COVID-19 severity^17^, ^18^, ^19^^,^^47^^,^^48^ (Figure S7). Furthermore, increased plasmablast frequencies in severe patients complemented prior reports^19^^,^^47^ (Figure S7). While changes in frequencies of monocyte subsets were not among the most informative features of our severity model, a secondary analysis focused on monocyte subsets recapitulated previously reported monocytic changes associated with disease severity, including increased frequency of classical monocytes, decreased frequency of non-classical monocytes, and decreased HLA-DR expression by (classical, non-classical) monocytes in severe patients (Figure S8).^16^, ^17^, ^18^^,^^22^^,^^47^^,^^49^, ^50^, ^51^ In particular, we observed a reduction in frequency of CD16^+^ non-classical monocytes, which was recently suggested to be mediated by antibody-mediated SARS-CoV-2 infection and pyroptosis.^52^

**Figure 4:**
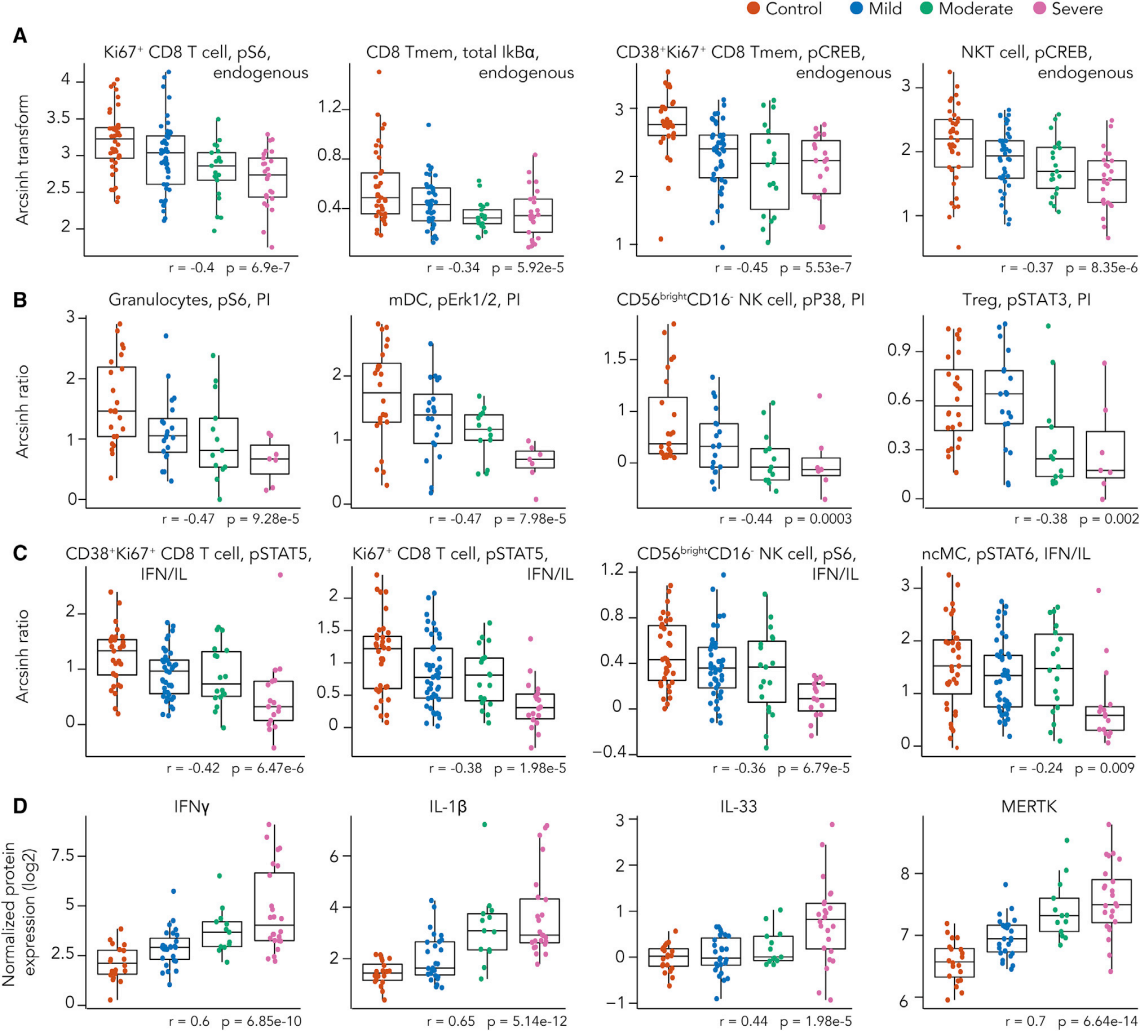
Severity model features reveal biological signatures that demarcate patients with mild, moderate, and severe COVID-19 Boxplots, classified by disease severity, showing features informative to the model. r and p indicate Spearman coefficient and p value of Spearman correlation of the feature with disease severity. (A) Endogenous immune cell signaling (arcsinh transformed values; see STAR Methods). (B) Immune cell signaling response to PI stimulation is reported as the arcsinh transformed ratio over the endogenous signaling response (see STAR Methods). (C) Immune cell signaling responses to IFNα/IL-2/IL-4/IL-6 (IFN/IL) stimulation are reported as the arcsinh transformed ratio over the endogenous signaling response (see STAR Methods). (D) Plasma protein levels are reported as the normalized protein expression, an arbitrary unit provided by the Olink assay. Tmem, memory T cell; MERTK, tyrosine-protein kinase Mer. For boxplots, the center line represents the median value; upper and lower box limits indicate first (Q1) and third (Q3) quartile, respectively; whiskers, minimum (Q1−1.5∗IQR) and maximum (Q3+1.5∗IQR). IQR, interquartile range. AUC, area under the curve. See also Figure S7–S11.

Assays examining endogenous and stimulation-dependent cell signaling markers revealed alterations in cell states associated with varying disease severity. A negative correlation was observed between endogenous signaling in CD8^+^ T cell subsets and natural killer T (NKT) cells and COVID-19 severity, notably for the pS6, total IkBα, and pCREB signals (Figures 4A and S9A). Negative correlations with COVID-19 severity were also observed for endogenous p4EBP1 and total IkBα signals in granulocytes, while endogenous granulocyte pERK1/2 signal was positively correlated with disease severity (Figure S9A). Overall, these results suggest that alterations in endogenous immune cell signaling markers exist between COVID-19-severity categories. In particular, endogenous signaling activities of key elements of the mTOR, MAPK, and NF-κB pathway (pS6, pERK1/2, pCREB, and total IkBα) were diminished with increasing disease severity in immune cell populations that play important roles in the defense against viral pathogens, such as CD8^+^ T, NKT, and granulocyte cell subsets.

Additionally, immune cell signaling responses to PI stimulation were largely diminished with increasing disease severity, especially for innate immune cell populations. Specifically, PI-stimulated signaling responses in granulocytes (pS6, pERK1/2, and pP38), myeloid dendritic cells (mDCs) (pERK1/2 and pSTAT3), and CD56^bright^CD16^-^ NK cells (pP38) were negatively correlated with disease severity (Figures 4B and S9B). Negative correlations with disease severity were also observed for the pSTAT3 signal in regulatory T cells (Tregs) and pMAPKAPK2 signal in CD161^+^CD8^+^ T cells in response to PI stimulation (Figures 4B and S9B). Decreased responsiveness to PI stimulation may be an indication of reduced effector function of circulating innate and adaptive immune cells during severe COVID-19, driven by cell-intrinsic effects and/or changes in cytokines and other modulating factors present in the circulation.

For immune cell signaling responses to IFNα/IL-2/IL-4/IL-6 stimulation, we also observed a negative correlation with disease severity for the pSTAT4/5/6 signals in both adaptive and innate immune cells (Figures 4C and S9C), suggestive of an impaired signaling response to IFN and cytokines in those individuals with more severe COVID-19. Indeed, others have also observed an impaired type I IFN activity in peripheral immune cells of patients critically ill with COVID-19, shown by downregulation of IFN-stimulated genes upon whole-blood IFNα stimulation.^53^

Among the most robust plasma proteomic features identified by the severity model were several features that positively correlated with disease severity and which overlapped with prior descriptions of the cytokine storm syndrome described in patients with severe COVID-19.^8^^,^^11^ For instance, plasma levels of the cytokines IFNγ, IL-1β, and IL-33, showed a positive correlation with disease severity (Figure 4D). IL-6, one of the first plasma cytokines recognized as elevated during COVID-19,^54^ also exhibited a positive correlation with disease severity in our cohort (Figure S10). Although this feature contributed to the severity model, it ranked well below the top 10% of bootstrap-selected informative features (Data S1). A similar positive correlation was observed for lung-related proteins in circulation, such as pulmonary surfactant-associated protein A2 (SFTPA2) and cathepsin H (CTSH), which are involved in surfactant homeostasis (Figure S10).^55^^,^^56^

Angiotensin-converting enzyme 2 (ACE2) is used as a viral entry receptor by SARS-CoV-2 and is released from the epithelial cell surface upon viral binding.^57^ ACE2 ranked within the top 10% of informative features, and levels showed a positive correlation with severity (Figure S10). The severity model also identified the protease MME (neprilysin), another key player of the renin-angiotensin system (RAS),^58^ as a positive correlate of COVID-19 severity (Figure S10). MME is also implicated in neutrophil degranulation,^59^ and our list of informative model features contained multiple other proteins involved in neutrophil degranulation (protein pathway identified by Reactome; see STAR Methods; Reactome gene set identifier R-HSA-6798695.2; Figure S11A), which were mainly positively correlated with severity as well. This dysregulation of neutrophil degranulation in severe patients is in agreement with recent plasma proteome findings.^60^

Our analysis revealed tyrosine-protein kinase Mer (MERTK) as the most robust feature in the proteome dataset contributing to the severity model (Data S1). Levels of MERTK, an immunosuppressive tyrosine kinase receptor,^61^ were positively correlated with disease severity (Figure 4D). MERTK is found on the surface of macrophages, where it mediates phagocytosis of apoptotic cells.^62^ Activation of MERTK has an immunosuppressive effect by downregulating the production of cytokines and type I IFNs.^61^^,^^62^ Increased MERTK shedding could result in reduced surface expression and loss of MERTK signaling,^63^ which could play a central role in the hyper inflammation observed in severe COVID-19. MERTK also plays a role in platelet aggregation^63^ and endothelial barrier integrity.^64^ Plasma MERTK levels were not positively correlated with most available markers of activation of monocyte subsets (Data S3). Plasma levels of several other proteins involved in primary hemostasis (Reactome gene set identifier R-HSA-109582) were also found to be informative in our model of disease severity (Figure S11B), with the majority of them displaying positive correlations with increasing disease severity.

To gain further insight into the dynamic behavior of plasma and immune cell events in patients with COVID-19, the most informative severity model features (top 10%) were correlated with time since symptom onset for each patient severity category and data layer (Figure S12; Data S1). Only 5 out of 374 model features examined showed correlations with p values below a false discovery rate (FDR)-adjusted value of 0.05, indicating that the large majority of model features were not significantly correlated with time since symptom onset in this dataset (Figure S12A; Data S1C). Among those 5 features with significant time association, ITIH3 and LGALS9 levels appear elevated near symptom onset in severe COVID-19 but decline precipitously thereafter, unlike more stable trajectories in mild and moderate patients (Figure S12B). These features may therefore have important diagnostic value in distinguishing severe cases soon after infection.

In summary, informative features of the severity model revealed biological signatures that progressed from mild to moderate and severe COVID-19. Salient characteristics of this biological progression included cellular elements of immune signaling networks implicated in defensive immunity against viral pathogens (such as the progressive dampening of NF-κB, MAPK/mTOR, and JAK/STAT signaling in multiple innate and adaptive immune cell subsets) and sentinel proteomic pathways involved in lung and RAS homeostasis, primary hemostasis, neutrophil degranulation, and inflammation.

## Discussion

This cross-sectional study combined high-content plasma proteomics with the single-cell analysis of immune signaling responses to identify biological determinants of severity across the spectrum of COVID-19 manifestations. Using a two-step analytical approach that accounts for the dimensionality of different data layers, we built and independently validated an integrated model that classifies COVID-19 severity. The biological underpinnings of the severity model consisted of coregulated plasma and single-cell proteomic elements that progressed with COVID-19 severity, including the inflammatory cytokine response to SARS-CoV-2, the mobilization of the RAS and primary hemostasis system, and the dysregulation of the JAK/STAT, NF-κB, and MAPK/mTOR immune signaling responses. The identification of biological signatures progressing with COVID-19 severity provides a set of sentinel events detectable in the early phase of infection that may be possible therapeutic targets for the prevention and/or treatment of severe COVID-19.

The ongoing pandemic has fueled major research efforts toward understanding the host immune response against SARS-CoV-2 infection.^8^^,^^13^^,^^15^, ^16^, ^17^^,^^19^ Previous efforts have been particularly focused on hospitalized patients with severe COVID-19, which, while of paramount importance, excludes the majority of SARS-CoV-2-infected patients who suffer from mild or moderate COVID-19 and do not require hospitalization. The comparative analysis of samples from patients with mild, moderate, and severe disease afforded a more exhaustive characterization of immune responses related to COVID-19 severity. Our approach dovetails with prior studies identifying an immunological shift distinguishing the spectrum of COVID-19-infection states.^18^^,^^21^ Consistent with our results, this switch included an increase in inflammation, the emergence of CD4 and CD8 T cells with a proliferative-exhausted phenotype, and a distinct activated myeloid signature.^18^^,^^21^ In previous work, immune cell function and responses were indirectly inferred through either phenotype or endogenous single-cell mRNA transcriptomics changes, while our approach provided a direct assessment of endogenous intracellular signaling responses of multiple immune cell subsets as well as their capacity to respond to inflammatory stimulation.

Two major biological signatures associated with the progression from mild to moderate and severe disease emerged from our integrated analysis: (1) the dampening of NF-κB, MAPK/mTOR, and JAK/STAT intracellular signaling responses in multiple innate and adaptive immune cell subsets, and (2) the mobilization of a proteomic network enriched for elements of the RAS, lung homeostasis, and hemostasis pathways, alongside canonical elements of the cytokine storm signature of severe COVID-19.

Several of the features informative to our severity model resonate with previous findings in patients suffering from severe COVID-19. For example, plasma levels of cytokines such as IFNγ, IL-1β, IL-33, and IL-6 increased with increasing severity, consistent with the cytokine storm observed in patients with severe COVID-19^8^^,^^11^^,^^14^ and with circulating IL-33 levels as a potential indicator of damaged lung tissue.^65^, ^66^, ^67^ In addition, changing frequencies of circulating immune cells also aligned with prior reports in severe patients.^15^^,^^17^^,^^19^^,^^47^^,^^48^ Interestingly, an increased percentage of granulocytes was observed in the PBMC fraction of patients with severe COVID-19.^15^^,^^16^^,^^18^^,^^68^^,^^69^ Low-density granulocytes also appear in the PBMC fraction of patients with inflammatory diseases and severe infection.^70^^,^^71^

In addition to previously reported features of COVID-19 disease severity, the functional analysis of intracellular signaling events in this study revealed intriguing new biology, notably with respect to immune cell responses to inflammatory ligands. The aforementioned low-density granulocytes can display dysfunctional immune responses,^70^^,^^71^ which supports our observation of a decreased immune cell signaling response by the granulocytes present in the PBMC fraction of patients with severe disease. In addition, in agreement with Overmyer et al.*,*^60^ increasing plasma levels of several proteins involved in neutrophil degranulation were correlated with disease severity. Excessive release of granules can result in tissue damage and is a feature of acute lung injury and septic shock.^72^ In addition to granulocytes, other cell types such as mDCs, NK cells, NKT cells, Tregs, and CD4 and CD8 T cells also showed an inverse relationship between capacity to respond to cytokine stimulation and disease severity, suggestive of overall diminished effector functions of circulating innate and adaptive immune cells with increasing severity. These results are consistent with several other studies that have shown decreased functional responses and exhausted phenotypes in peripheral innate and adaptive immune cells in severe patients as well.^15^^,^^17^^,^^20^^,^^53^^,^^73^^,^^74^ In this dataset, dampened immune cell responsiveness observed in severe patients was not correlated with time since symptom onset. These effects may represent a prolonged feature of severe disease itself, a dampened pre-infection state, or underlying genetic susceptibility.^75^^,^^76^ Furthermore, observations of dampened mTOR or JAK/STAT signaling in innate and adaptive immune cells could be informative for potential treatment recommendations for COVID-19, as drugs can have different influences on signaling responses in innate and adaptive immune cells. For instance, methylprednisolone administration to patients undergoing surgery inhibits JAK/STAT signaling responses in the adaptive compartment only.^25^ As such, restoring the effector responses of circulating immune cells by immune potentiators (e.g., immune checkpoint inhibitors) to enhance host immunity while simultaneously controlling the cytokine storm (e.g., corticosteroids) may be beneficial in preventing severe disease and overcoming infection.^77^, ^78^, ^79^

In addition to dysregulated immune signaling responses, the examination of the severity model features revealed several key proteomic pathways that were mobilized with disease severity, including pathways related to lung homeostasis, RAS homeostasis, and hemostasis. Notably, the plasma levels of two proteins implicated in the production of lung surfactant (SFTPA2 and CTSH^55^^,^^56^) markedly increased with disease severity. Both SFTPA2 and CTSH are synthesized in type II pneumocytes,^55^^,^^56^ which are primary targets for SARS-CoV-2 infiltration. As such, these proteins may be early markers of type II pneumocyte dysfunction, impaired surfactant synthesis, and lung damage, as SFTPA2 plasma levels are elevated in patients suffering from acute respiratory failure.^80^ These results are consistent with recent transcriptomics analyses of lung biopsies showing impaired surfactant production in patients with severe COVID-19.^81^ Elevated SFTPA2 and CTSH could be important markers for clinicians to differentiate severe patients and stratify those who may be most at need of respiratory support.

Key proteomic features of the COVID-19 severity model also included two components of the RAS (ACE2 and MME), a complex hormonal system that regulates blood pressure and fluid homeostasis as well as pulmonary inflammation.^58^ The involvement of the RAS in the pathogenesis of SARS-CoV-2 infection is well established, since the interaction between the viral spike protein and ACE2 is a primary mechanism of viral entry into host cells.^57^ High ACE2 and MME plasma levels in severe patients suggest increased shedding from the cell surface, which may result in loss of function of these proteins.^82^ A loss of function of ACE2 and MME will likely result in a decrease in angiotensin I (Ang I) and Ang II degradation, which aligns with the multi-organ injuries observed in severe patients.^82^^,^^83^ Interestingly, increased plasma ACE2 concentration is associated with an increased risk for subsequent cardiovascular events in patients with COVID-19.^84^ As such, our analysis points at mechanistic markers of disease severity that may also be implicated in the clinical manifestations of patients recovering from COVID-19, such as cardiovascular or neurological complications.

The most informative protein feature in our severity model was soluble (s) MERTK, for which plasma levels increased with severity. sMERTK can be produced by proteolytic ectodomain shedding of membrane-bound (mb) MERTK, a member of the Tyro-Axl-MerTK (TAM) family of receptor tyrosine kinases, the activation of which leads to immunosuppression and macrophage-mediated apoptotic cell phagocytosis.^61^, ^62^, ^63^^,^^85^ sMERTK can act as a competitive inhibitor of MERTK signaling by sequestering ligands that could otherwise bind to mbMERTK.^61^, ^62^, ^63^ In the context of COVID-19, impaired MERTK signaling has been proposed as a link between the hyperinflammatory and hypercoagulative state observed in patients with severe disease.^61^^,^^86^, ^87^, ^88^ In the model proposed by Lemke et al., sequestration of MERTK-ligand protein spike (S) by developing blood clots results in impaired MERTK signaling in neighboring macrophages and increased pro-inflammatory cytokine production.^61^ Sequestration of protein S by sMERTK may also contribute to this effect. In addition, loss of endothelial mbMERTK has been shown to exacerbate lung inflammation in the context of acute respiratory distress syndrome by enhancing endothelial permeability and leukocyte transendothelial migration at the site of infection.^64^ The pleiotropic roles of MERTK in the regulation of lung endothelial integrity, coagulation, and inflammation suggest that impaired MERTK signaling may be a central component of the pathogenesis of severe COVID-19. sMERTK levels were not positively correlated with markers of monocyte activation in this study, raising the possibility that sMERTK could instead be driven by other factors such as pyroptosis of circulating monocytes,^52^ by activation of monocyte/macrophages in the tissues, or by loss of endothelial mbMERTK in the lung. Strategies to regulate ADAM-17-dependent shedding of mbMERTK may be worth investigating in the context of COVID-19.^89^, ^90^, ^91^

Assessing the dynamic behavior of model features (top 10%) over the course of disease showed that, consistent with our confounder analysis, the majority of features of the severity model (98.66%) did not correlate with time since symptom onset. The levels of only 5 single-cell or plasma proteomic features correlated with the timing of symptom onset. Notably, ITIH3 and LGALS9 (galectin-9) levels were elevated early after symptom onset in severe patients and declined over the course of disease, suggesting that these proteomic features may be important early indicators of patients with severe disease. Elevated plasma galectin-9 levels have been proposed to be a contributor to the cytokine storm observed in SARS-CoV-2-infected patients,^92^ while serum ITIH3 levels have been found to be more abundant in fatal COVID-19 cases upon intensive care unit (ICU) admission, with levels decreasing over the course of disease in both survivors and non-survivors.^93^

Determining the underlying immune pathogenesis across the spectrum of COVID-19 severity remains an important clinical challenge. Our integrated analysis of plasma and single-cell proteomics in patients with mild, moderate, and severe COVID-19 identified a multi-variate model that differentiates COVID-19 severity. The observations identified by this model contribute clinically relevant insights into the status of patient’s immune responses during SARS-CoV-2 infection and provide promising severity-specific biological signatures for future validation that may inform decision-making on potential therapeutic targets for the prevention of disease progression.

### Limitations of the study

This study has certain limitations. First, in this cohort, we only assessed peripheral blood samples of patients affected by COVID-19. If available from future cohorts, it would be highly informative to assess the local immune and proteome perturbations in parallel by analyzing lung biopsies or bronchoalveolar lavage fluid to investigate whether the same trends and dampened immune cell responses are observed locally in the lung as well. Second, there are discrepancies in the definition of COVID-19-severity categories—especially for the definition mild—that can hinder the comparison of different studies. In this study, we defined mild patients as those SARS-CoV-2-infected patients that only experience mild symptoms without any breathing issues and that do not require hospitalization,^33^ which is similar to the work done by Chevrier et al.^18^ and Silvin et al*.*^22^ Others, on the other hand, defined mild as those SARS-CoV-2-infected patients that were hospitalized but had no or only low oxygen requirements.^16^^,^^94^, ^95^, ^96^ Despite these differences, the tremendous effort of the research community to rapidly make research available is an enormous advantage for much-needed fast-paced research into COVID-19, and the availability of public datasets will empower future meta-analyses. Thirdly, while it is becoming clear that those severe patients that die versus those that survive can be phenotypically distinct,^39^ the presence of only 3 non-survivors in our cohort precludes an adequately powered analysis to decouple severity-related factors from survival-related factors. Fourthly, our study is cross-sectional in nature. While a time-dependent representation of cross-sectional data provides insight into disease evolution, serial blood sample collection and longitudinal molecular monitoring will be necessary in future studies to discern cause versus effect and identify predictive features that precede severe COVID-19 disease or the development of post-acute sequelae. While several longitudinal studies have indeed shown that fluctuations exist over the disease course,^39^^,^^97^, ^98^, ^99^, ^100^, ^101^ these studies have mainly been conducted in hospitalized patients, and a longitudinal study into the disease dynamics of non-hospitalized patients with only mild and/or moderate disease has not been studied rigorously. Finally, while the results of the plasma proteomic model were validated in an independent cohort from a different center,^39^ further validation with orthogonal methods (i.e., ELISA, flow cytometry, etc.) will be imperative in the development of future diagnostic tests.

## STAR★Methods

### Key resources table

**Table:** 

REAGENT or RESOURCE	SOURCE	IDENTIFIER
**Antibodies**		

Anti-human 4E-BP1 pT37/46 (clone 236B4)	CST	Cat# 2855; RRID: AB_560835
Anti-human BDCA3 (clone 1A4)	BD Biosciences	Cat# 559780; RRID: AB_397321
Anti-human CCR7 (clone 150503)	R&D Systems	Cat# MAB197-100; RRID: AB_2072803
Anti-human CD11b (clone ICRF44)	BioLegend	Cat# 301302; RRID: AB_314154
Anti-human CD11c (clone Bu15)	BioLegend	Cat# 337202; RRID: AB_1236381
Anti-human CD123 (clone 7G3)	BD Biosciences	Cat# 554527; RRID: AB_395455
Anti-human CD14 (clone M5E2)	BioLegend	Cat# 301802; RRID: AB_314184
Anti-human CD16 (clone 3G8)	BioLegend	Cat# 302033; RRID: AB_2104002
Anti-human CD161 (clone HP-G310)	BioLegend	Cat# 339902; RRID: AB_1501090
Anti-human CD19 (clone J3-119)	Beckman Coulter	Cat# IM1313; RRID: AB_131613
Anti-human CD1c (clone AD5-8E7)	Miltenyi	Custom Carrier Free; RRID: AB_244309
Anti-human CD235a (clone HIR2)	BioLegend	Cat# 306602; RRID: AB_314620
Anti-human CD27 (clone O323)	BioLegend	Cat# 302802; RRID: AB_314294
Anti-human CD3 (clone SP34.2)	BD Biosciences	Cat# 551916; RRID: AB_394293
Anti-human CD33 (clone AC104.3E3)	Miltenyi	Custom Carrier Free; RRID: AB_615078
Anti-human CD38 (clone HIT2)	BioLegend	Cat# 303502; RRID: AB_314354
Anti-human CD4 (clone OKT4)	BioLegend	Cat# 317402; RRID: AB_571963
Anti-human CD45 (clone HI30)	BioLegend	Cat# 304002; RRID: AB_314390
Anti-human CD45RA (clone HI100)	BioLegend	Cat# 304102; RRID: AB_314406
Anti-human CD56 (clone NCAM16.2)	BD Biosciences	Cat# 559043; RRID: AB_397180
Anti-human CD61 (clone VI-PL2)	BioLegend	Cat# 336402; RRID: AB_1227584
Anti-human CD66 (clone YTH71.3)	Pierce	Cat# MA1-36189; RRID: AB_1073288
Anti-human CD7 (clone M-T701)	BD Biosciences	Cat# 555359; RRID: AB_395762
Anti-human CD8 (clone RPA-T8)	BioLegend	Cat# 301002; RRID: AB_314120
Anti-human CREB pS133 (clone 87G3)	CST	Cat# 9198; RRID: AB_2561044
Anti-human Erk1/2 pT202/Y204 (clone D13.14.4E)	CST	Cat# 4370; RRID: AB_2315112
Anti-human FoxP3 (clone PCH101)	Thermo Fisher	Cat# 14-4776-82; RRID: AB_467554
Anti-human HLA-DR (clone Immu357)	Beckman Coulter	Cat# Immu357; RRID: AB_131284
Anti-human IgM (clone G20-127)	BD Biosciences	Cat# 555780; RRID: AB_396115
Anti-human IkBa amino-terminal (clone L35A5)	CST	Cat# 4814; RRID: AB_390781
Anti-human Ki67 (clone SolA15)	Thermo Fisher	Cat# 14-5698-82; RRID: AB_2688057
Anti-human MAPKAPK2 pT334 (clone 27B7)	CST	Cat# 3007; RRID: AB_490936
Anti-human P38 pT180/Y182 (clone 36/p38)	BD Biosciences	Cat# 612289; RRID: AB_399606
Anti-human PLCγ2 pY759 (clone K86–689.37)	BD Biosciences	Custom Carrier Free; RRID: AB_647226
Anti-human S6 pS235/236 (clone 2F9)	CST	Cat# 4856; RRID: AB_2181037
Anti-human STAT1 pY701 (clone 4a)	BD Biosciences	Cat# 612233; RRID: AB_399555
Anti-human STAT3 pY705 (clone 4)	BD Biosciences	Cat# 612357; RRID: AB_399646
Anti-human STAT4 pY693 (clone 38)	BD Biosciences	Cat# 612738; RRID: AB_399957
Anti-human STAT5 pY694 (clone 47)	BD Biosciences	Cat# 611965; RRID: AB_399386
Anti-human STAT6 pY691 (clone 18)	BD Biosciences	Cat# 611597; RRID: AB_399013
Anti-human TBK1/NAK pS172 (clone D52C2)	CST	Cat# 5483; RRID: AB_10693472
Anti-human Zap70/Syk pY319/Y352 (clone 17a)	BD Biosciences	Cat# 612574; RRID: AB_399864

**Chemicals, peptides, and recombinant proteins**		

RPMI 1640 medium, no glutamine	Gibco	Cat# Fisher 21870092
Penicillin-Streptomycin	Gibco	Cat# Fisher 15140122
L-Glutamine	Gibco	Cat# Fisher 25030081
Fetal Bovine Serum (FBS)	Gibco	Cat# Fisher 16140071
PBS	Gibco	Cat# Fisher 14190250
LPS	Invivogen	Cat# Fisher TLRLPEKLPS
CL097	Invivogen	Cat# Fisher NC1203867
IFNa	Invitrogen	Cat# Fisher PI111012
IL2	R&D Systems	Cat# Fisher 202IL010CF
IL4	R&D Systems	Cat# Fisher 204IL010CF
IL6	R&D Systems	Cat# 206IL010CF
PI	Invitrogen	Cat# Fisher 00497503
BSA	Sigma Aldrich	Cat# A3059-50G
Sodium Azide	Sigma Aldrich	Cat# S2002-25G

**Critical commercial assays**		

Smart Tube Proteomic Stabilizer	Smart Tube Inc.	Cat# PROT1
FcBlock: Human TruStain FcX	Biolegend	Cat# 422302
100% Methanol	Thermo Fisher	Cat# 50-980-487
Iridium DNA Intercalator	Fluidigm	Cat# 201192B
16% Paraformaldehyde	Thermo Fisher	Cat# 50-980-487
Four Element Normalization Beads	Fluidigm	Cat# 201078

**Deposited data**		

Raw and processed data	Dryad	https://doi.org/10.5061/dryad.9cnp5hqmn

**Software and algorithms**		

Cell Engine	Primity Bio	https://cellengine.com
Single Cell Debarcoder	Nolan Lab	https://github.com/nolanlab/single-cell-debarcoder
Bead Normalization	Nolan Lab	https://github.com/nolanlab/bead-normalization
MOFA2	Argelaguet et al.^34^	v1.0
Mgcv	Wood^102^	v1.8-31

**Other**		

CyTOF 2 mass cytometer	Fluidigm	N/A

### Resource availability

#### Lead contact

Further information and requests for resources and reagents should be directed to an will be fulfilled by the lead contact, Brice Gaudillière (gbrice@stanford.edu).

#### Materials availability

This study did not generate new unique reagents.

### Experimental model and subject details

#### Study design

This study was designed as a cross-sectional study, where samples were obtained from adults with positive test results for SARS-CoV-2 from analysis of nasopharyngeal swab specimens. Samples were obtained at any point from March to June 2020 at the Stanford Occupational Health Clinic. Testing was accomplished using Stanford Health Care clinical laboratory developed internal testing capability with a quantitative reverse-transcriptase–polymerase-chain-reaction (qRT-PCR) assay. Mechanical ventilation and therapeutics for COVID-19 (when used) were not administered until after collection of blood sample, with the exception of 2 individuals who were put on mechanical ventilation one day prior to sample collection (information in supplemental file “PatientCharacteristics”). Patients were excluded from enrollment if they were taking experimental therapeutics for COVID-19 (i.e. those medications not authorized by a regulatory agency for use in COVID-19). Healthy controls were collected prior to the detection of SARS-CoV-2 in the region (historical controls). Figure S4 shows self-reported symptom onset and diagnostic qRT-PCR timing in relation to the day of sample collection.

#### Data sources and clsinical definitions

We obtained data from self-reported surveys and from Stanford clinical data electronic medical record system as per consented participant permission. This database contains all the clinical data available from Stanford facilities. The data obtained included patients’ demographic details, vital signs, laboratory test results, medication administration data, historical and current medication lists, historical and current diagnoses, time of COVID-19 symptom onset (self-reported), clinical notes, radiological results, biopsy results as appropriate, historical discharge disposition for previous inpatient hospitalizations, and ventilator use data. Severity diagnosis was assigned at time of SARS-CoV-2 qRT-PCR alone. Severity diagnosis was assigned according to previously defined National Institute of Health (NIH) criteria^32^^,^^33^ with the clinical classification of COVID-19 as follows; Asymptomatic: Positive for SARS-CoV-2 but without COVID-19 symptoms; Mild disease: Various mild symptoms (e.g. cough, fever, sore throat, loss of smell and taste, etc.) but no breathing issues (shortness of breath, dyspnea, or abnormal chest imaging) are reported; Moderate disease: Evidence of lower respiratory tract disease but oxygen saturation (SpO_2_) ≥ 94%; Severe disease: Requires hospitalization because of respiratory distress (SpO_2_ ≤ 94%, respiratory frequency <30 breaths/min, PaO2/FiO2 <300 mm Hg, or lung infiltrates >50%).

#### Phlebotomy and initial blood processing

Blood was collected from 97 patients positive for SARS-CoV-2 and 40 controls via venipuncture. Anticoagulated blood was processed into peripheral blood mononuclear cells (PBMC) by density gradient centrifugation using published methods.^103^ PBMC were stored in 10% DMSO and frozen in liquid nitrogen until thawing and staining. Plasma was isolated from blood collected in EDTA tubes as follows: Within 4hrs of collection, tubes were centrifuged at 500 x g for 10 min at room temperature (RT), plasma was transferred into fresh conical tubes and centrifuged again (500 x g, 10 min, RT) before aliquoting into 500 μL cryovials and transferred to −80°C for long-term storage.

#### Study approval

We conducted this study at Stanford University Medical Center, where the samples from COVID-19 patients were collected at the Stanford Occupational Health Clinic under an IRB approved protocol (55,689; Protocol Director Dr. Nadeau). Informed consent was obtained from each patient prior to enrolling in the study or from the patient’s legally authorized representative if the patient was unable to provide consent. Healthy controls (historical controls) were consented using a separate IRB-approved protocol (8629; Protocol Director Dr. Nadeau).

### Method details

#### Mass cytometry analysis of single-cell immune cell responses in PBMC

##### *In vitro* PBMC stimulation

Cryopreserved PBMC were quickly thawed, washed two times with supplemented medium, and rested for 1h at 37°C in RPMI 1640 medium supplemented with 10% fetal bovine serum, 1% Penicillin-Streptomycin, and 1% L-Glutamine. PBMC were counted and checked for viability. 0.5-1x10^6^ cells were either stimulated with lipopolysaccharide (LPS; 1 μg/ml) and CL097 (TLR7/8 agonist; 1 μg/ml), interleukin-2 (IL-2), IL-4, IL-6 and interferon-*a* (IFN*a*; all 100 ng/mL), a cocktail of phorbol 12-myristate 13-acetate (PMA), ionomycin, brefeldin A and monensin (1x; PI cocktail), or left unstimulated for 15 min at 37°C. After stimulation, samples were fixed with Proteomic Stabilizer (SmartTube) and stored at −80°C until further processing for mass cytometry analysis.

##### Barcoding and antibody staining

The 42-marker mass cytometry antibody panel included 25 cell surface antibodies and 17 intracellular antibodies recognizing primarily phospho-specific signaling epitopes (Data S2). In brief, following *in vitro* stimulation, fixed PBMCs were thawed, reconstituted in cell staining medium, and arranged in a 96-well block. Subsequent steps were performed using a previously described robotics platform.^104^ Sets of 16 samples were barcoded with palladium metal^105^ and pooled into a single well. Pooled barcoded samples were treated with FC-block (Human TruStain FcX, Biolegend) for 10 min then surface antibody stained for 30 min in cell staining medium (PBS with 0.5% BSA and 0.02% sodium azide). After surface staining, cells were permeabilized in ice-cold 100% methanol, washed, and stained for 60 min with intracellular antibodies in cell staining medium. Following intracellular staining, cells were washed and resuspended in an iridium intercalator (Fluidigm) solution containing 1.6% paraformaldehyde. Finally, samples were washed, resuspended in 1X five-element normalization beads (La, Pr, Tb, Tm, Lu) (Fluidigm), and analyzed on a freshly cleaned and tuned CyTOF instrument. The resulting mass cytometry data were bead-normalized across all runs and debarcoded as previously described.^106^

##### Minimization of experimental batch effects

To minimize potential batch effects, each of the unstimulated and stimulated samples from the same individual were processed, barcoded, stained, and measured simultaneously in the same tube. Samples were processed, barcoded, stained, and measured simultaneously in batches equalized to the extent possible for age, gender, healthy control, mild, moderate, and severe categories. Internal controls -- i.e. aliquots of the same sample -- were added during each mass cytometer run to evaluate consistent performance between runs. To control for consistent tuning parameters of the mass cytometer, batches of samples measured on separate days were normalized to metal impregnated beads mixed with samples during runs.

##### Cell frequency, endogenous intracellular signaling, and intracellular signaling responses

Mass cytometry data was examined using CellEngine (Primity Bio) to define cell populations using manual gates and quantify differential expression of signaling markers in response to stimulation. The gating strategy and representative example of phosphosignaling responses can be found in Figure S1 and S2.

Cell frequencies were expressed as a percentage of gated singlet mononuclear cells (DNA^+^CD235a^−^CD61^−^CD66^−^), except for granulocyte frequency which was expressed as a percentage of singlet leukocytes (DNA^+^CD235a^−^CD61^−^). Signal intensity was quantified per single cell for each phospho-signaling protein (pSTAT1, pSTAT3, pSTAT4, pSTAT5, pSTAT6, pMAPKAPK2, pCREB, pPLCγ2, pS6, pERK1/2, pP38, pZAP70/Syk, pTBK1, p4EBP1, and total IkB*a*) and for a set of markers that relate to immune subset specific functionality (HLA-DR, FoxP3, CD38, IgM, and Ki67) using an arcsinh transformed value (arcsinh(x/5)) from the median signal intensity. Endogenous intracellular signaling activity was derived from the analysis of unstimulated cells, while intracellular signaling responses to stimulation were reported as the arcsinh transformed ratio over the endogenous signaling, i.e., the difference in arcsinh transformed signal intensity between the stimulated and unstimulated condition. For cell subsets in a given sample that had an event count below 20 events, that cell subset and related phospho-signal were excluded from downstream analysis. A combination of five stimulations x 44 immune cell subsets x 20 functional proteins (i.e. 15 phospho-signaling + 5 functionality-specific proteins) resulted in a total of 4400 single features obtained per sample. A penalization matrix, based on mechanistic immunological knowledge, was applied to the immune cell response data,^35^^,^^107^ resulting in a final total of 2662 features for each sample that was used for further analysis. These included 44 innate and adaptive immune cell subset frequency features, 789 endogenous signaling features, 299 LPS/CL097 stimulation response features, 752 IFN*a*/IL-2/IL-4/IL-6 stimulation response features, and 778 PI stimulation response features.

##### Plasma protein profiling using olink multiplex panel

Plasma protein levels were quantified using Olink multiplex proximity extension assay (PEA) panels (Olink Proteomics; www.olink.com) according to the manufacturer’s instructions and as described before.^108^ The basis of PEA is a dual-recognition immunoassay, where two matched antibodies labeled with unique DNA oligonucleotides simultaneously bind to a target protein in solution. This brings the two antibodies into proximity, allowing their DNA oligonucleotides to hybridize, serving as a template for a DNA polymerase-dependent extension step. This double-stranded DNA which is unique for a specific antigen will get amplified using P5/P7 Illumina adapters along with sample indexing, which is quantitatively proportional to the initial concentration of target protein. These amplified targets will finally get quantified by Next Generation Sequencing using Illumina Nova Seq 6000 (Illumina Corporation. San Diego, California). In this study, we have used the Explore 1536 panel which measures 1,472 proteins using 3 μL plasma samples, which were treated with 1% Triton X-100 and incubated at room temperature for 2 h to inactivate the virus.

The raw expression values obtained with the Olink assay are provided in the arbitrary unit Normalized Protein Expression (NPX), where high NPX values represent high protein concentration. Values were log2-transformed to account for heteroskedasticity. Proteins close to the limit of detection are flagged in the raw data.

#### Benchmarking of models

In addition to the LASSO model for which we built the stack generalization model (see “Multivariate analysis and stacked generalization”), we also compared the performance of the LASSO regression with different regression strategies, including: ordinary least square regression, random forest regressor, elastic-net regression, and support vector machine regression. For all these methods, we report (1) the RMSE (root-mean-square-error) obtained through the leave-one-out cross validation (LOOCV) strategy and (2) the RMSE on the validation set (Table S3).

#### MGH dataset validation

We used a recent proteomic study^39^ of patients enrolled at the Mass General Hospital (MGH, Boston, MA) as an independent validation cohort of the severity model trained on the plasma proteomic data. For this analysis, we used our previous proteomic model fitted on our training cohort to predict disease severity and compared to the WHO scale established for those samples at day 0 to reproduce the hypothesis of our model. We report the r and p value of the Spearman correlation test between the predictions of our model and the WHO scale labels (Related to Figure S6 and Data S1). As the MGH dataset was missing 43 protein expressions compared to the assay that we used, we imputed the median value of the training cohort to samples of the MGH dataset before predicting.

### Quantification and statistical analysis

#### Univariate analysis

We used the R environment (http://www.r-project.org/) for statistical analysis. We chose to apply a ranked regression analysis for each feature relative to the severity of the patient at the time of sampling using a spearman correlation test. Healthy controls were labeled with the numerical value 1, mild cases with 2, moderate = 3, and severe = 4. For each statistic, we report both p values associated with the spearman correlation coefficient, and the r correlation coefficient.

#### Multi-omic factor analysis

Multi-omic factor analysis (MOFA) was applied simultaneously across plasma (Olink) and the single-cell proteomics data (cell frequency, endogenous signaling, IFN*a*/IL-2/IL-4/IL-6 signaling response, LPS/CL097 signaling response, and PI signaling response). MOFA infers a set of factors that capture both biological and technical sources of variability that are shared across different datasets. MOFA models were constructed using the six data layers which were supplied as a list of matrices. We followed the developers’ directions for model selection and downstream analysis.^34^ Since MOFA is not guaranteed to find a global optimum, 10 model trials were performed using different random initializations. For each trial, the number of factors was calculated by requiring at least 2% variance explained for any single dataset. The model with the highest evidence lower bound was selected out of these 10 trials (model 6, Figure S3A). The factors calculated by model six of our 10 trials were extracted for downstream analysis. MOFA enables variance decomposition of the calculated factors and uses a coefficient of determination (R^2^) to quantify the fraction of variance explained by each factor for each dataset, which we examined first to determine how each dataset contributes to each factor (Figure S3B). Next, we regressed the 17 factors on COVID-19 severity, which was dummy encoded as follows: Control = 1, Mild = 2, Moderate = 3, Severe = 4. Regression estimates, 95% confidence intervals, and p values were examined (Figure S3C). Factors 3, 8, and 10 were significantly associated with COVID-19 severity. To assess the robustness of factors across model trials, we calculated Pearson correlation coefficients between every pair of factors across all trials. Factors 3 and 10 were consistently discovered in all model instances (data not shown). Finally, we visualized our samples across factors 3 and 10 using a bivariate scatterplot (Figure 1D).

#### Covariate analysis

Correlations between the covariates “age”, “gender”, “obesity”, “Hispanic ethnicity”, and “days between self-reported symptom onset and sample collection” with our outcome “severity” was assessed with Pearson correlation and are reported as a correlation heatmap (Figure S5). Gender was encoded as ‘Male’ = 0, ‘Female’ = 1. Obesity was encoded as ‘not obese’ = 0, ‘obese’ = 1. Hispanic ethnicity was encoded as ‘not Hispanic’ = 0, ‘Hispanic’ = 1.

#### Multivariate analysis and stacked generalization

For the multivariate analysis, a LASSO model was trained independently on each omics dataset independently using the caret and glmnet packages. For a matrix X of all biological features from a given -omic dataset of N samples, and a vector of disease severity Y, the LASSO algorithm (29) calculates coefficients β to minimize the error term $L\left(\beta \right)\;=\;\frac{1}{N}{\left(Y\;-\;XB\right)}^{2}\;+\;\lambda {\left||B|\right|}_{1}\;$. The L1 regularization is used to increase model sparsity for the sake of biological interpretation and model robustness. Once a LASSO model is trained for each omics modality (individual model performance Table S2), the multi-omics analysis can be carried out by performing stacked generalization^35^ on the new representation of the data by using the outputs of the previous layer of models as predictors with the constraint that their participation should be a non-negative coefficient. Specifically, a LASSO model is first constructed on each omics modality, then all estimations of disease severity are used as predictors for a second-layer constrained regression model. Intrinsically, this is equivalent to a weighted average of the individual models with the coefficients of the LASSO model as desired weights.

#### Leave-one-out cross-validation

An underlying assumption of the LASSO algorithm is statistical independence between all observations. At each iteration of this algorithm, one sample is kept for independent validation. This is an extreme case of the k-fold cross validation called leave-one-out cross validation (LOOCV).^109^ The model is trained on all the samples except the one blinded sample and the reported results are exclusively based on the blinded subject. We repeat this method for every sample, hence building N successive models. For stacked generalization, a cross-validation was implemented in a two-step approach where the first layer selects the best values of λ for each individual omic and reports the intermediate prediction. Then, the second layer optimizes the constrained regression of all predictors for the stacked generalization step.

The results of the training cohort were validated with an independent cohort that is totally blinded during the design and fitting of the models. This independent validation is used only for performance evaluation throughout the results and used to assess the performance as a second step after the cross-validation.

#### Multi-class receiver operating characteristics (AUC)

To characterize our predictions' separability in a multi-class setting, we used a combined metric of the area under the receiver operating curve (AUC) in both the training and validation model. This metric for multi-class analysis uses every combination of labels in one-to-one comparisons. Every subset of predictions on a pair of classes is considered independently, and the AUC is calculated for each specific pair. Once all the subset AUCs are computed, the generalizable AUC combines them, taking their mean across all comparisons, to give the multi-class model’s overall performance.^110^

#### Bootstrap analysis for feature selection

For each omics dataset, we performed a bootstrap analysis where we repeat a bootstrapping procedure on the dataset and train a cross-validated model. At each iteration, we keep the non-zero coefficients selected by the LASSO procedure on the bootstrapped dataset and we repeat the procedure 1,000 times.^45^^,^^46^ We report the frequency of selection of the features as well as their median coefficient in all the bootstrap models. This method selected 44 frequency, 599 endogenous, 536 PI-response, 492 IFN*a*/IL-2/IL-4/IL-6, and 783 proteome features that were informative in at least one iteration of the bootstrap procedure (see Data S1). To assess the relative importance of each feature to the model, we ranked features in each data layer based on their frequency of selection.

#### Correlation network

The features are visualized using a correlation graph structure to identify correlated feature populations. We used a tSNE^111^ layout for the visualization of all the features calculated from the matrix including all the samples available. On this graph, each biological feature is denoted by a node whose size is dependent on the *-*log_*10*_ of p value of correlation with disease severity (Spearman). The correlation network between the features is represented by an edge where the width of the edges is proportional to the Spearman p value of the correlation between a pair of nodes on a log10 scale.

#### Confounder analysis

A post-hoc linear regression analysis was used as a statistical method to exclude the likelihood that certain clinical or demographic variables confounded the predictive accuracy of the severity model. We considered the following confounders: “days between symptoms onset and sample collection”, “age”, “gender”, “Hispanic ethnicity”, and “obesity”. This analysis fits a regression using the values of the cross-validated SG model and adds the confounders together to regress them against the severity predictor. The resulting coefficients of the regression are computed and reported in Table S4. Their significance is reported using the F-statistic from which we derive the p value for the model coefficient and the confounders.

#### Plasma proteomic pathway identifier

To obtain pathway information of selected proteome features, we utilized Reactome (www.reactome.org), a web-based resource for identifying biological pathways, in which we used the list of the 10% bootstrap-selected proteins as an input. Reactome provided a list of pathways, identified by a Reactome gene set identifier, which we assessed for the proteins part of specific pathways.

#### Longitudinal modeling

Correlation analysis of bootstrap selected features (10%) with “time since symptom onset” were calculated with a generalized additive model (GAM) using the R package “mgcv”.^102^ For each feature, a factor-smooth interaction GAM was constructed with Severity and “time since symptom onset” as explanatory variables. Three knots were used to represent the smooth term. P-values of smooth terms for Mild, Moderate, and Severe groups were corrected for false discovery per data layer with Benjamini-Hochberg,^112^ and are reported in Data S1C.

Model formula (R notation) y ∼ Severity + s(DOS, by = Severity, k = 3).

## Data Availability

Raw data (FCS file), original data (Olink plasma proteomic data), processed data (cell frequency, phosphosignal, and chord diagram correlation matrix data), patient characteristics, and Generalized Additive Model (GAM) p values have been deposited to Dryad: https://doi.org/10.5061/dryad.9cnp5hqmn. Supplemental files are available from Mendeley Data: https://doi.org/10.17632/xss9pdtc4f.1. Source code used for analysis can be found on github at https://github.com/julienhed/COVID-Severity. Any additional information required to reanalyze the reported study is available upon request from the lead contact.

## Supplemental information

Document S1. Figures S1–S12 and Tables S1–S4

Data S1. Bootstrap-selected features, related to Figures 3, 4 and S7–S11, comparison of severity correlation of plasma proteome features with Filbin et al.^39^ dataset, related to Figure S6, and results of GAM model, related to Figure S12

Data S2. Mass cytometry antibody panel, related to Figures 1, S1, and S2

Data S3. Correlation of sMERTK levels and available markers of monocyte activation (Spearman correlation), related to Figure 4

Document S2. Article plus supplemental information
