# Supplementary material for: Integrated plasma proteomic and single-cell immune signaling network signatures demarcate mild, moderate, and severe COVID-19
Source: Cell Rep Med. 2022 Jun 28;3(7):100680. doi: 10.1016/j.xcrm.2022.100680 (PMC9238057; doi:10.1016/j.xcrm.2022.100680)
Supplement: Document S1. Figures S1–S12 and Tables S1–S4 [file mmc1.pdf]

Cell Reports Medicine, Volume *3*

Supplemental information

Integrated plasma proteomic and single-cell immune signaling network signatures demarcate mild, moderate, and severe COVID-19

Dorien Feyaerts, Julien Hédou, Joshua Gillard, Han Chen, Eileen S. Tsai, Laura S. Peterson, Kazuo Ando, Monali Manohar, Evan Do, Gopal K.R. Dhondalay, Jessica Fitzpatrick, Maja Artandi, Iris Chang, Theo T. Snow, R. Sharon Chinthrajah, Christopher M. Warren, Richard Wittman, Justin G. Meyerowitz, Edward A. Ganio, Ina A. Stelzer, Xiaoyuan Han, Franck Verdonk, Dyani K. Gaudillière, Nilanjan Mukherjee, Amy S. Tsai, Kristen K. Rumer, Danielle R. Jacobsen, Zachary B. Bjornson-Hooper, Sizun Jiang, Sergio Fragoso Saavedra, Sergio Iván Valdés Ferrer, J. Daniel Kelly, David Furman, Nima Aghaeepour, Martin S. Angst, Scott D. Boyd, Benjamin A. Pinsky, Garry P. Nolan, Kari C. Nadeau, Brice Gaudillière, and David R. McIlwain

Supplementary information

Integrated plasma proteomic and single-cell immune signaling network signatures demarcate mild, moderate, and severe COVID-19

Dorien Feyaerts, Julien Hédou, Joshua Gillard, Han Chen, Eileen S. Tsai, Laura S. Peterson, Kazuo Ando, Monali Manohar, Evan Do, Gopal K.R. Dhondalay, Jessica Fitzpatrick, Maja Artandi, Iris Chang, Theo T. Snow, R. Sharon Chinthrajah, Christopher M. Warren, Rich Wittman, Justin G. Meyerowitz, Edward A. Ganio, Ina A. Stelzer, Xiaoyuan Han, Franck Verdonk, Dyani K. Gaudillière, Nilanjan Mukherjee, Amy S. Tsai, Kristen K. Rumer, Danielle R. Jacobsen, Zach B. Bjornson, Sizun Jiang, Sergio Fragoso Saavedra, Sergio Iván Valdés Ferrer, J. Daniel Kelly, David Furman, Nima Aghaeepour, Martin S. Angst, Scott D. Boyd, Benjamin A. Pinsky, Garry P. Nolan, Kari C. Nadeau, Brice Gaudillière, David R. McIlwain

Correspondence to: gbrice@stanford.edu (B.G., lead contact), gnolan@stanford.edu (G.P.N.)

This file includes:

Figs. S1 to S12
Tables S1 to S4

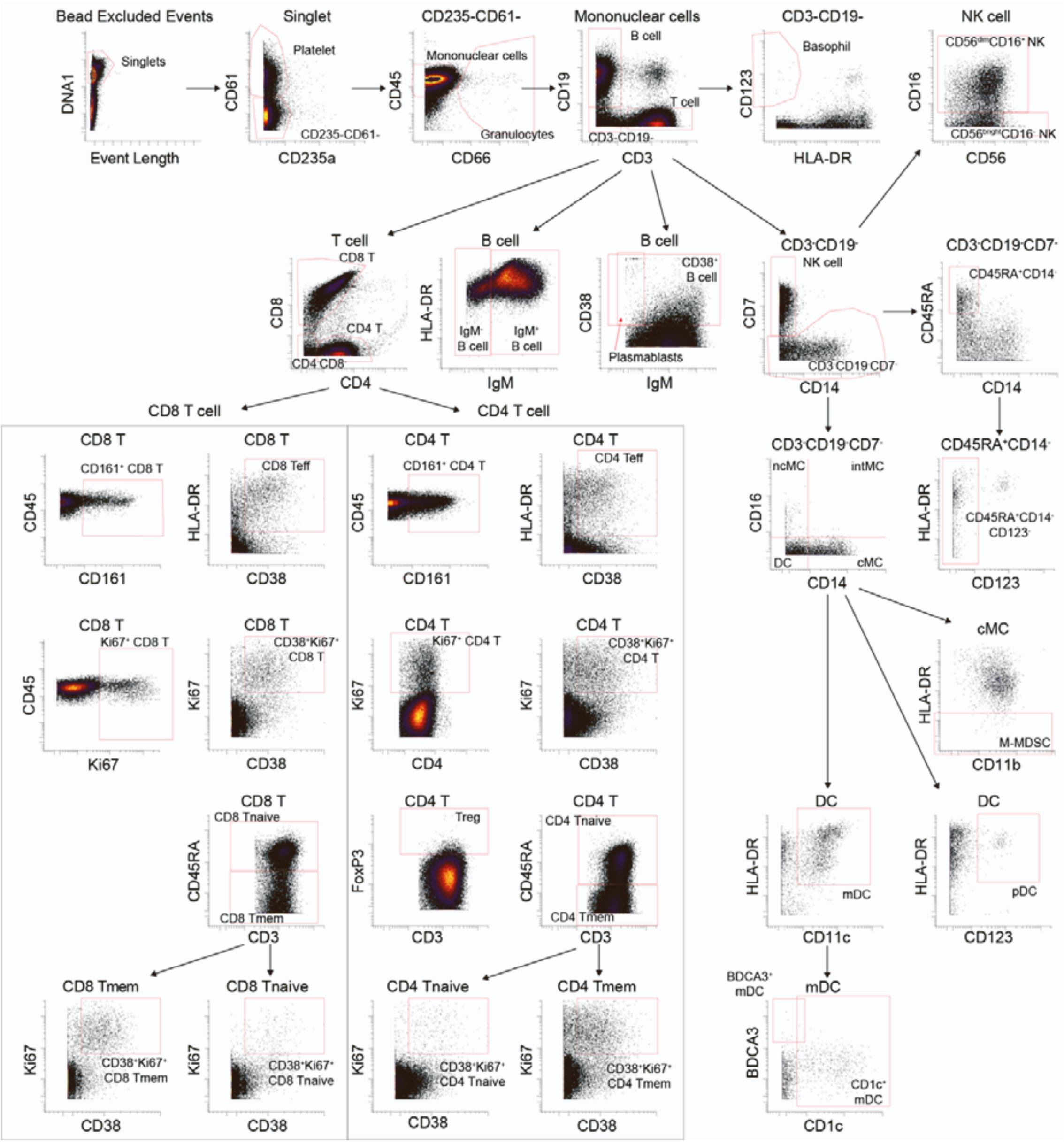

Supplemental Figure 1. Hierarchical manual gating strategy used for mass cytometry analysis. Related to Figure 1. Bivariate dot plots for identification of immune cell populations from peripheral blood mononuclear cells (PBMC). Gating strategy for one representative PBMC sample is shown.

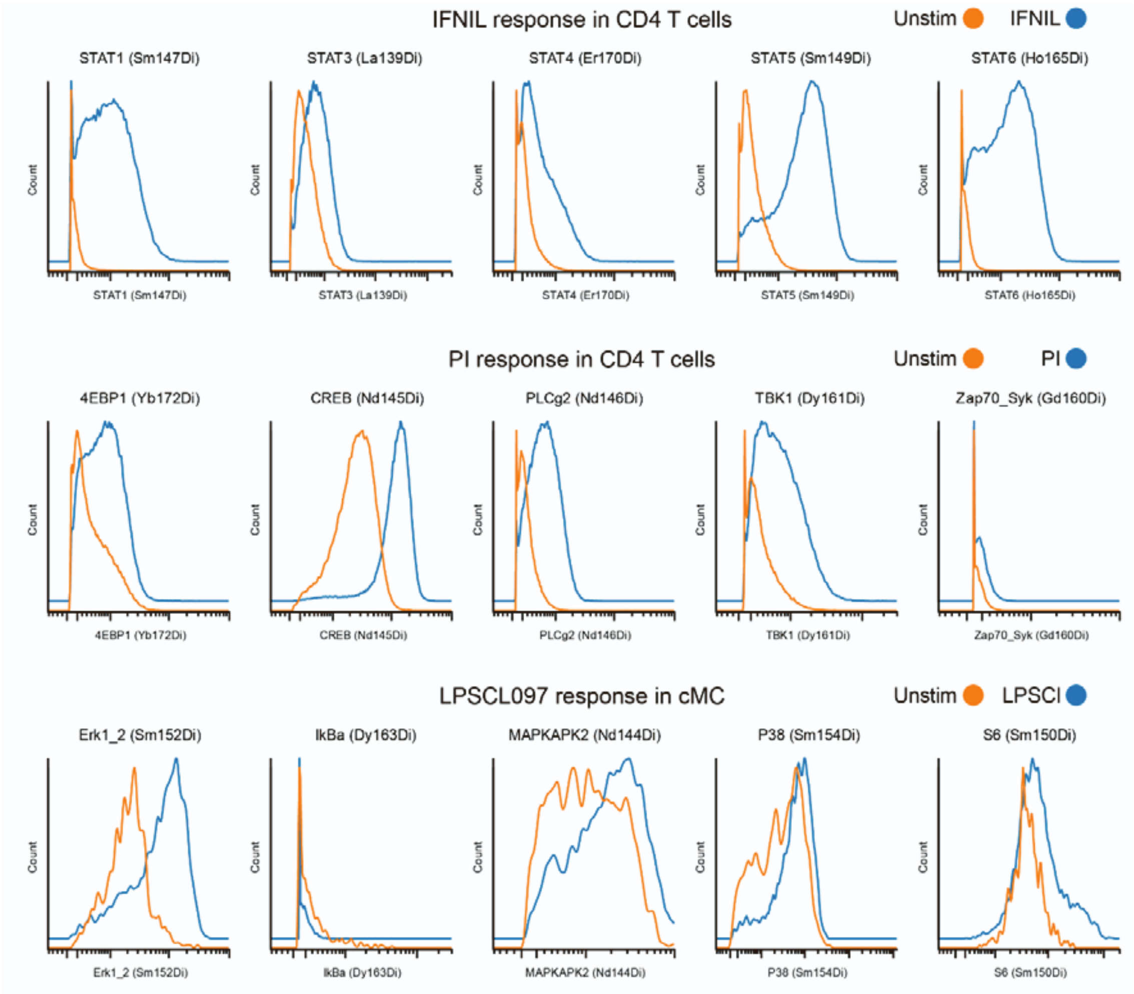

Supplemental Figure 2. Specific phosphosignaling responses. Related to Figure 1. Representative histograms for one representative PBMC sample is shown showing representative phosphosignal levels for unstimulated (orange lines) and stimulated (blue lines) cells. Row headings indicate stimulus (IFN/IL = IFNα/IL-2/IL-4/IL-6; PI = PMA+Ionomycin; LPSCL097 = LPS + CL097) and cell type ($CD4^+$ T cells and classical monocytes (cMC)).

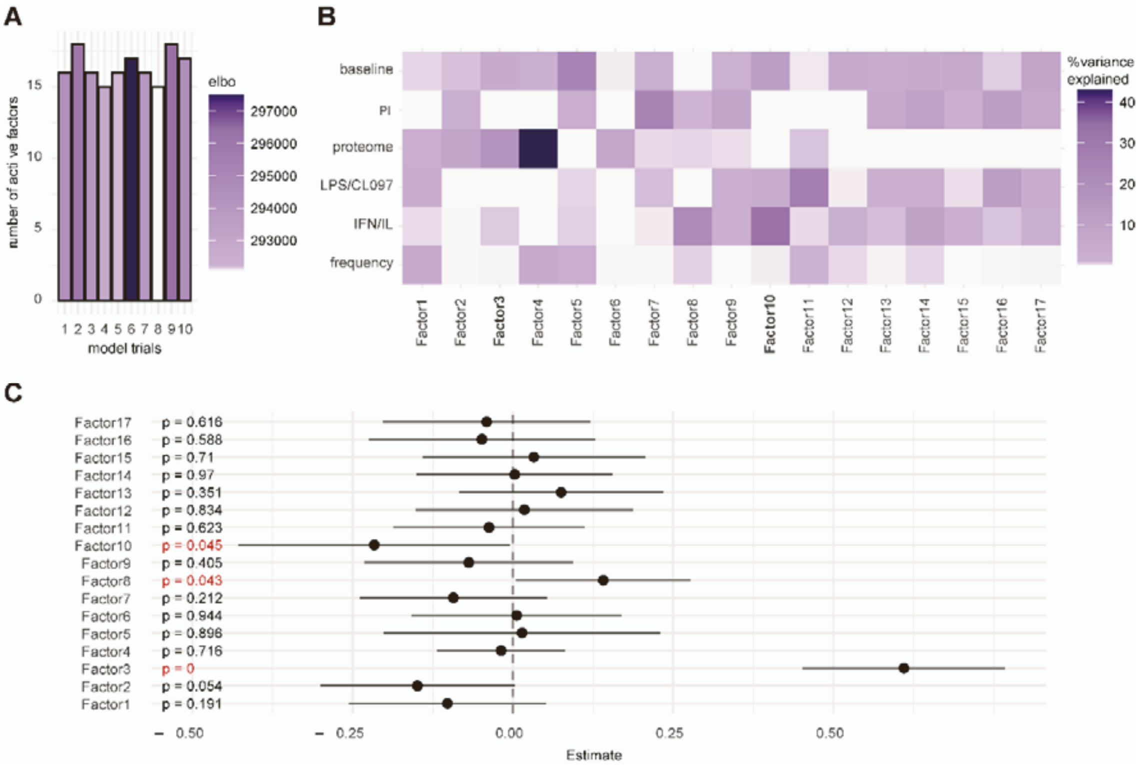

Supplemental Figure 3. Multi-omic factor analysis (MOFA) identifies shared sources of variability across single-cell cytometry and proteomic data that are associated with COVID-19 severity. Related to Figure 1. **A** Ten MOFA models were constructed with different random initializations. Shown are the number of factors calculated for each trial and colored by the corresponding evidence lower bound (ELBO). Model trial 6 is the subject of Figure S2B-C and Figure 1E. **B** Portion of variance explained (R^2) for each factor and for each dataset. IFN/IL = IFNα/IL-2/IL-4/IL-6. **C** Association of MOFA factors with COVID-19 severity (encoded as Control = 1, Mild = 2, Moderate = 3, Severe = 4) was performed with linear regression analysis. Estimates, 95% confidence intervals, and p-values are shown (significant p-values highlighted red).

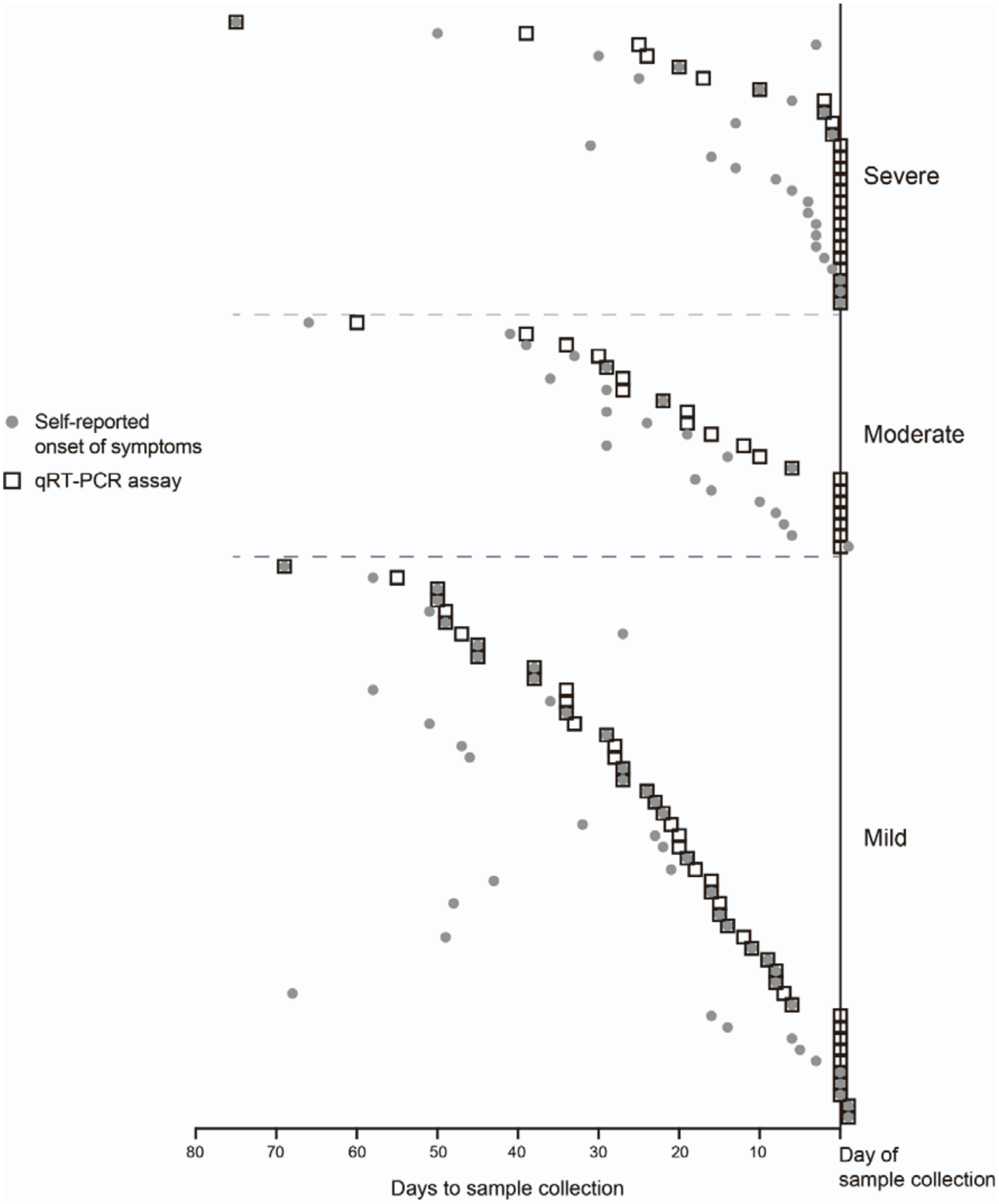

Supplemental Figure 4. Timing of sample collection in relation to self-reported symptom onset and day diagnostic SARS-CoV-2 qRT-PCR was performed. Related to Figures 1 and 2.

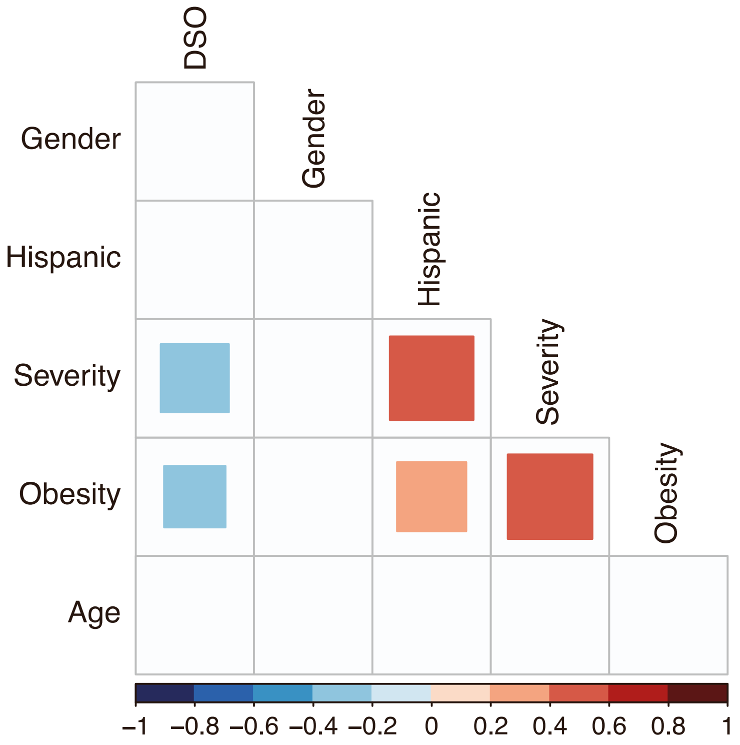

Supplemental Figure 5. Correlation analysis of covariates with severity. Related to Figures 1 and 2. Pairwise correlation heatmap of clinical and (socio)demographic variables. Only Pearson correlations coefficients with p-value <0.05 are shown. Heatmap color indicates Pearson correlation coefficient. DSO = Days Since Onset (days between reported symptom onset and sample collection.

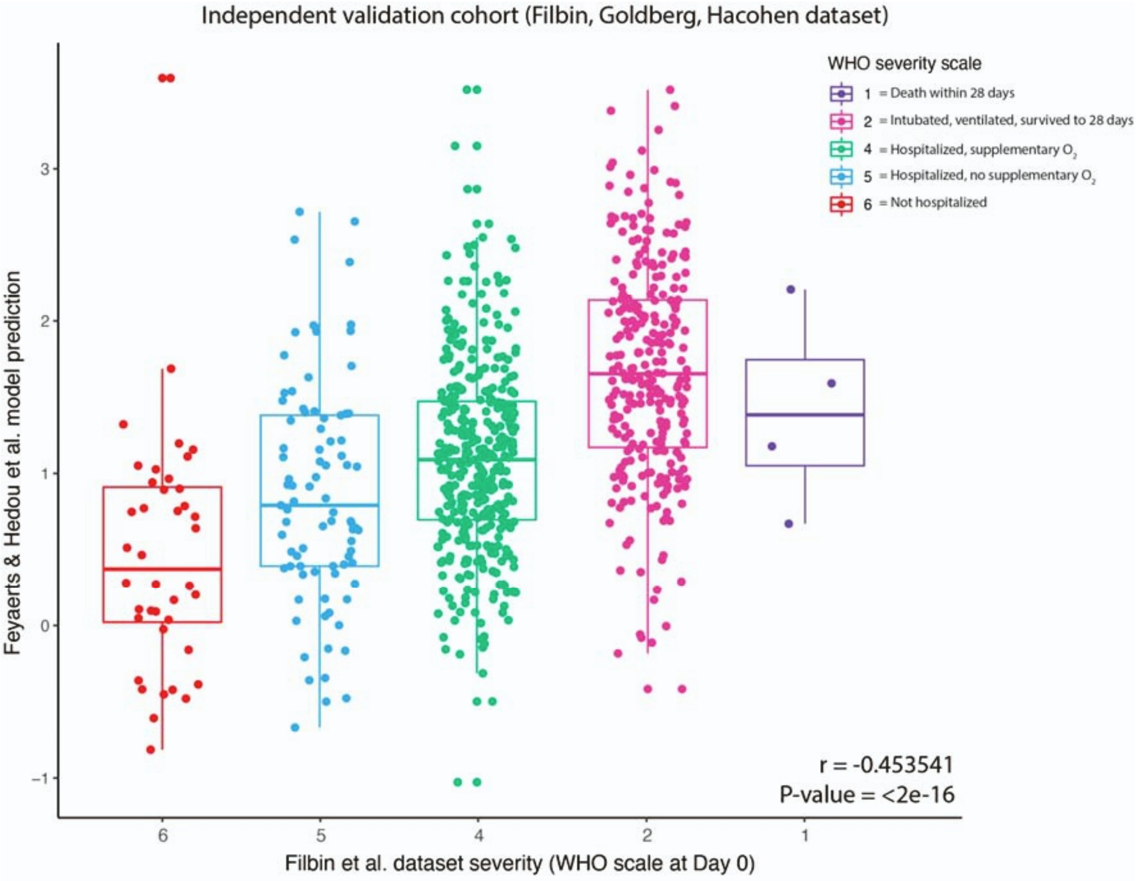

Supplemental Figure 6. Validation of COVID-19 severity model in an independent cohort provided by the MGH Emergency Department COVID-19 Cohort (Filbin, Goldberg, Hacohen) with Olink Proteomics. Related to Figure 2. COVID-19 severity model built on the Stanford training dataset and tested on an independent cohort reported by Filbin *et al.*[1]. Plot shows predicted vs true disease severity (WHO scale at Day 0). WHO scale used by Filbin *et al.*[1] is as follows: 1 = Death within 28 days; 2 = Intubated, ventilated, survived to 28 days; 4 = hospitalized, supplementary 0_2; 5 = Hospitalized, no supplementary 0_2; 6 = Not hospitalized. Spearman correlation coefficient for correlation of the model prediction with severity (WHO1 to WHO6) and corresponding p-values are shown on the plots. For boxplots, the center line represents the median value; upper and lower box limits indicate first (Q1) and third (Q3) quartile, respectively; whiskers, minimum (Q1-1.5*IQR) and maximum (Q3 + 1.5*IQR). IQR, interquartile range.

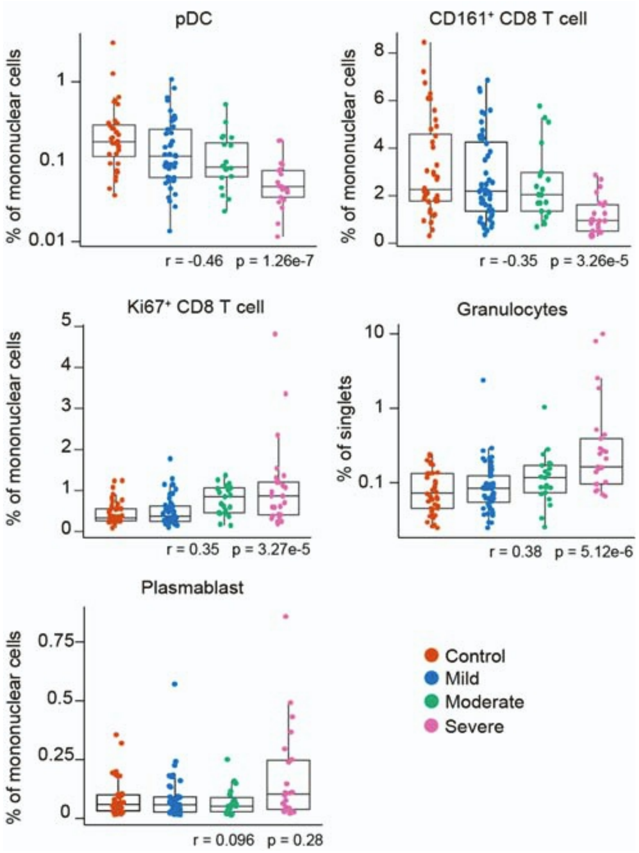

Supplemental Figure 7. Frequency features informative to the severity model. Related to Figures 3 and 4. Frequency features are shown as a percentage of mononuclear cells. Granulocytes are shown as a percentage of singlets. pDC and granulocyte frequency are plotted on a log-scale. Plasmablast were defined as $CD38^{+}IgM^{-}$ $CD19^{+}$. Boxplots classified by disease severity and including Spearman coefficient and corresponding p-value of correlation of the feature with disease severity. For boxplots, the center line represents the median value; upper and lower box limits indicate first (Q1) and third (Q3) quartile, respectively; whiskers, minimum (Q1-1.5*IQR) and maximum (Q3 + 1.5*IQR). IQR, interquartile range.

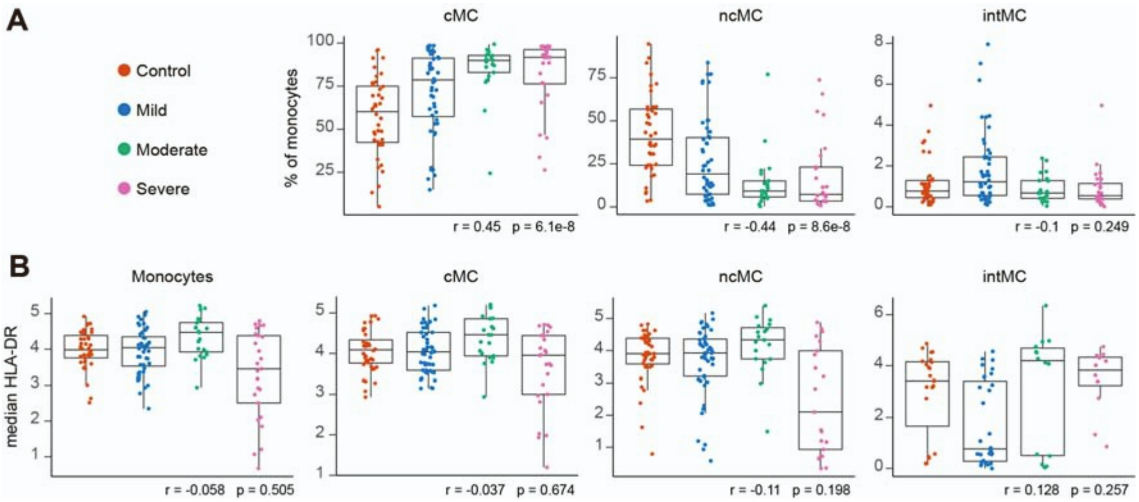

Supplemental Figure 8. Altered monocyte subset frequencies and HLA-DR expression in severe COVID-19 patients. Related to Figures 3 and 4. **A** Boxplots showing frequencies of monocyte subsets, plotted as a percentage of total monocytes. **B** Boxplots showing median HLA-DR expression by monocytes subsets, including Spearman coefficient and corresponding p-value of correlation of the feature with disease severity. Medians are reported as arcsinh transformed values. For boxplots, the center line represents the median value; upper and lower box limits indicate first (Q1) and third (Q3) quartile, respectively; whiskers, minimum (Q1-1.5*IQR) and maximum (Q3 + 1.5*IQR). IQR, interquartile range. cMC = classical monocyte; ncMC = non-classical monocyte; intMC = intermediate monocyte.

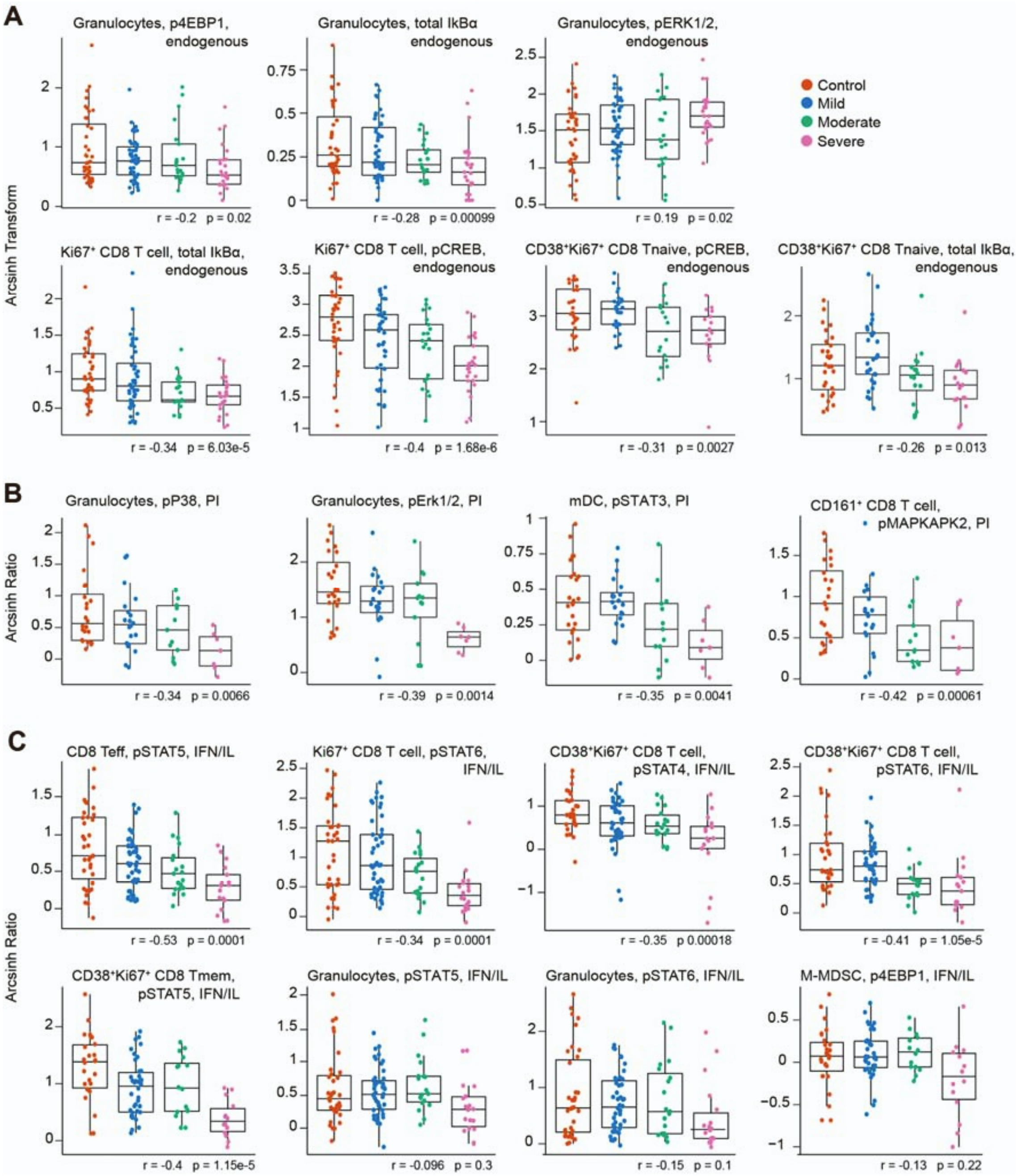

Supplemental Figure 9. Immune cell signaling response features informative to the severity model. Related to Figures 3 and 4. Boxplots showing endogenous immune cell signaling responses in unstimulated cells (reported as arcsinh transformed values), and signaling response after stimulation with inflammatory agents (reported as arcsinh transformed ratio over the endogenous (unstimulated) signaling response; see methods). **A** Endogenous immune cell signaling response. **B** immune cell signaling response to PI stimulation, and **C** response to IFNα/IL-2/IL-4/IL-6 stimulation (IFN/IL) of features informative in the COVID-19 severity model. Boxplots classified by disease severity and including Spearman coefficient and corresponding p-value of correlation of the feature with disease severity. For boxplots, the center line represents the median value; upper and lower box limits indicate first (Q1) and third (Q3) quartile, respectively; whiskers, minimum (Q1-1.5*IQR) and maximum (Q3 + 1.5*IQR). IQR, interquartile range. Tmem = memory T cell; Teff = effector T cell.

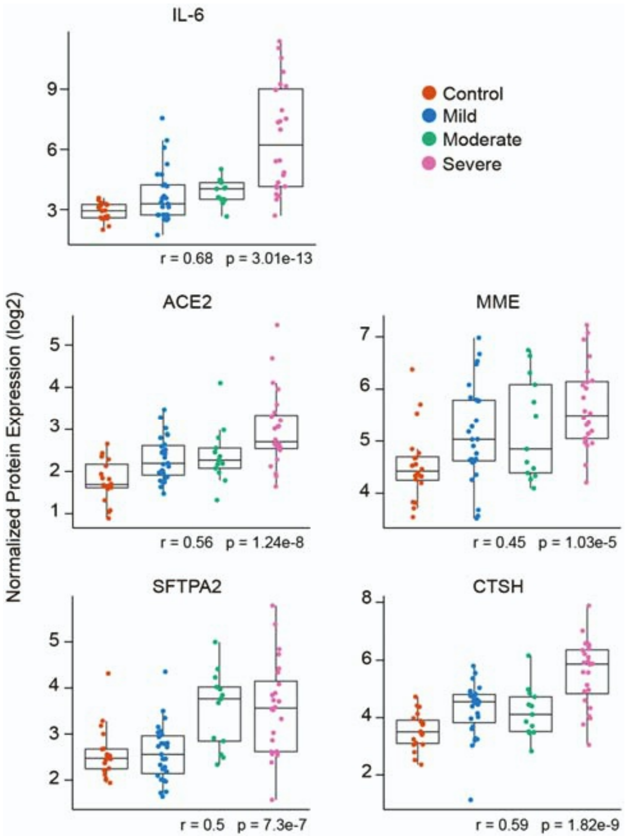

Supplemental Figure 10. Plasma proteomic features informative to the severity model. Related to Figures 3 and 4. Boxplots showing plasma protein levels of features that are informative in the COVID-19 severity model. Boxplots classified by disease severity and including Spearman coefficient and corresponding p-value of correlation of the feature with disease severity. For boxplots, the center line represents the median value; upper and lower box limits indicate first (Q1) and third (Q3) quartile, respectively; whiskers, minimum (Q1-1.5*IQR) and maximum (Q3 + 1.5*IQR). IQR, interquartile range. ACE2 = angiotensin-converting enzyme 2; MME = neprilysin; SFTPA2 = surfactant-associated protein A2; CTSH = cathepsin H.

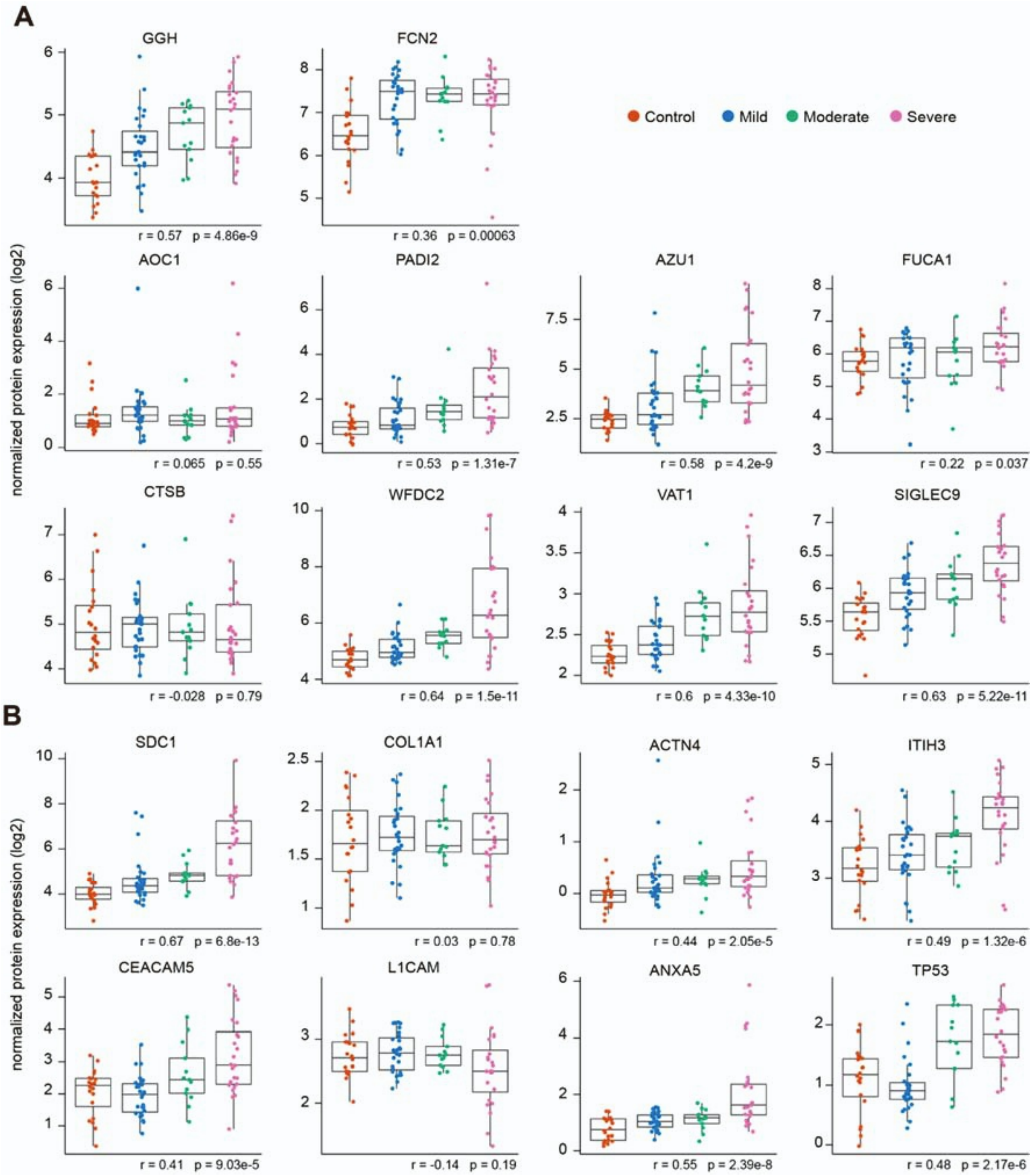

Supplemental Figure 11. Informative plasma proteomic features involved in neutrophil degranulation and hemostasis. Related to Figures 3 and 4. Boxplots showing plasma protein levels of features that are informative in the COVID-19 severity model and that are part of **A** neutrophil degranulation (Reactome gene set identifier R-HSA-6798695.2) and **B** hemostasis (Reactome gene set identifier R-HSA-109582). Boxplots classified by disease severity and including Spearman coefficient and corresponding p-value of correlation of the feature with disease severity. For boxplots, the center line represents the median value; upper and lower box limits indicate first (Q1) and third (Q3) quartile, respectively; whiskers, minimum (Q1-1.5*IQR) and maximum (Q3 + 1.5*IQR). IQR, interquartile range. GGH = Gamma-glutamyl hydrolase; FCN2 = Ficolin-2; AZU1 = Azurocidin; FUCA1 = Tissue alpha-L-fucosidase; AOC1 = Amiloride-sensitive amine oxidase [copper-containing]; PADI2 = Protein-arginine deiminase type-2; VAT1 = Synaptic vesicle membrane protein VAT-1 homolog; SIGLEC9 = Sialic acid-binding Ig-like lectin 9; CTSB = Cathepsin B; WFDC2 = WAP four-disulfide core domain protein 2; SDC1 = syndecan-1; COL1A1 = Collagen alpha-1(I) chain; ACTN4 = Alpha-actinin-4; ITIH3 = Inter-alpha-trypsin inhibitor heavy chain H3; CEACAM5 = Carcinoembryonic antigen-related cell adhesion molecule 5; L1CAM = Neural cell adhesion molecule L1; ANXA5 = Annexin A5; TP53 = Cellular tumor antigen p53.

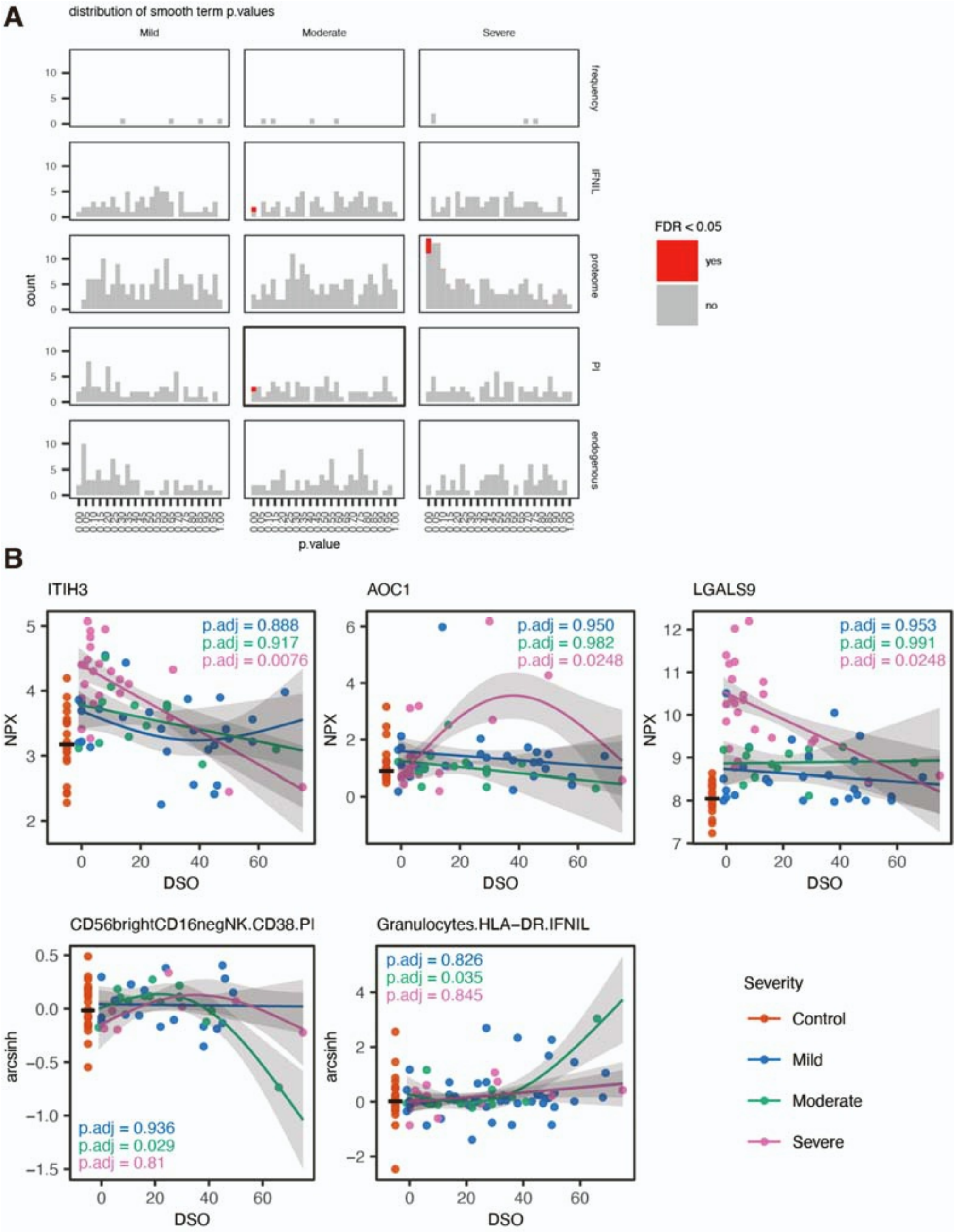

Supplemental Figure 12. Longitudinal representation of plasma proteomic and single cell immune signatures over the course of COVID-19 disease. Related to Figures 3 and 4. Longitudinal modeling was performed using a general additive model (GAM) with severity and time since symptom onset (DOS = Days Since Onset; days between reported symptom onset and sample collection) as explanatory variables on top 10% bootstrap selected features. **A** Density histograms of p-value distribution shows uniform p-value distribution in most groups and data layers except for the proteomic data layer in severe patients. Features that pass FDR adjusted p-values < 0.05 are colored red. **B** Features that passed FDR adjusted p-value < 0.05 are plotted according to DOS. On the graphs, lines indicate smooth term, grey area indicates confidence intervals, and p.adj are FDR adjusted p-values of the smooth term for a given severity class from the GAM model.

Data layer	training AUC	validation AUC	Weighted average of model coefficients of SG model
Frequency	0.7539	0.7896	0.12
Endogenous	0.7962	0.5907	0.43
LPS/CL097	0.5212	0.6117	0
IFNα/IL-2/IL-4/IL-6	0.5762	0.6099	0.008
PI	0.5784	0.8469	0.18
Proteome (Olink)	0.924	0.8362	0.73
Stacked Generalization model	0.799	0.7731	NA

Supplemental Table 1 – Model performance and weighted average of individual data layers. AUC (area under the curve) indicates the model performance of individual data layers in classifying severity. Weighted average shows the contribution of individual data layers to the severity model. Results related to Figure 2 B-C. SG model = Stacked Generalization model

A	training AUC	validation AUC
1vs2	0.71	0.6071
1vs3	0.85	0.7697
1vs4	0.90	0.9238
2vs3	0.71	0.6623
2vs4	0.82	0.8571
3vs4	0.81	0.8182
**B**		
1+2+3vs4	0.85	0.87
2+3vs4	0.82	0.85

Supplemental Table 2 – AUC of individual ROC analyses for performance of model to classify severity. Related to Figures 2 and S6. **A** AUC (area under the curve) shows how well the severity model is able to classify each patient group from the other. Results related to Figure 2 D-E. **B** Model performance to classify severe patients from other patient groups. 1 = control, 2 = mild, 3 = moderate, and 4 = severe.

RMSE obtained through LOOCV strategy (training set).						
**Regression models**	**LPS**	**PI**	**Endogenous**	**Frequency**	**IFN**	**Proteome**
**Lasso**	0.95795	1.01344	0.91424	0.99362	1.09569	0.64538
**EN**	1.04527	1.01955	0.99437	1.00308	1.09412	0.61143
**Ridge**	1.12907	1.09257	1.03075	1.05448	1.13382	0.65859
**RF**	1.11304	0.96971	1.12284	1.03347	1.06854	0.66897
**KNN**	1.18055	1.10033	1.17684	1.16178	1.14477	0.88511
**MLP**	1.33794	1.21787	1.29636	1.26883	1.17799	1.35069
**SVMlinear**	1.19477	1.17774	1.06828	1.53615	1.37531	0.65684
RMSE obtained on validation set						
**Regression models**	**LPS**	**PI**	**Endogenous**	**Frequency**	**IFN**	**Proteome**
**Lasso**	1.06401	0.86531	1.37507	0.97905	0.9194	1.03319
**EN**	1.05312	0.86343	1.41439	0.95212	0.90335	0.98736
**Ridge**	0.94008	0.87224	0.94515	1.74137	0.89918	0.78016
**RF**	0.95259	1.19001	0.8568	0.81054	1.00989	0.60655
**KNN**	1.00192	1.00881	0.88933	0.9775	0.9067	0.86262
**MLP**	0.99211	0.973	0.93151	2.35395	1.04441	1.37302
**SVMlinear**	1.14957	0.94113	1.25429	3.15656	1.03858	0.82602

Supplemental Table 3 – Benchmarking of models. Related to Figure 2 and Methods. Comparison of performance of LASSO regression with different regression strategies. Table shows RMSE obtained through the LOOCV strategy on the training set, and RMSE on the validation set. RMSE = root-mean-square error; EN = Elastic Net; Ridge = Ridge regression; RF = Random Forrest; KNN = k-nearest neighbor; MLP = multilayer perceptron; SVMlinear = support-vector machine linear regression; LASSO = least absolute shrinkage and selection operator; LOOCV = leave-one-out cross validation

Variable	Estimate	Std.Error	t value	Pr(>|t|)	
Prediction model	0.525444	0.139692	3.761	0.000961	***
Days between symptom onset and sample collection	-0.015960	0.006338	-2.518	0.018873	*
Age	0.001044	0.006054	0,172	0.864578	NS
GenderM	-0.377225	0.189705	-1.988	0.058278	<0.1
ObesityYes	-0.006722	0.234002	-0.029	0.977320	NS
HispanicEthnicityYes	0.409501	0.222364	1.842	0.077929	<0.1

Supplemental Table 4 – Confounder analysis. Related to Figures 2 and S5. Variables examined for potential confounding do not create a major impact on the COVID-19 severity model. Related to Figure 2. GenderM = male gender. NS = not significant. Significance codes: ‘*’ < 0.05, ‘***’ < 0.001.

Supplemental references

1. Filbin MR, Mehta A, Schneider AM, Kays KR, Guess JR, Gentili M, Fenyves BG, Charland NC, Gonye ALK, Gushterova I, et al. Longitudinal proteomic analysis of severe COVID-19 reveals survival-associated signatures, tissue-specific cell death, and cell-cell interactions. Cell Rep Med. 2021;2(5):100287.
